# Robust Characterization of Multidimensional Scaling Relations between Size Measures for Business Firms

**DOI:** 10.3390/e23020168

**Published:** 2021-01-29

**Authors:** Yuh Kobayashi, Hideki Takayasu, Shlomo Havlin, Misako Takayasu

**Affiliations:** 1 Department of Mathematical and Computing Science, School of Computing, Tokyo Institute of Technology, Yokohama 226-8502, Japan; kobayashi.y.bz@m.titech.ac.jp; 2 Institute of Innovative Research, Tokyo Institute of Technology, Yokohama 226-8502, Japan; takayasu@csl.sony.co.jp (H.T.); havlin@ophir.ph.biu.ac.il (S.H.); 3 Sony Computer Science Laboratories, Tokyo 141-0022, Japan; 4 Department of Physics, Bar-Ilan University, Ramat-Gan 52900, Israel

**Keywords:** business firms, companies, econophysics, multivariate scaling, nontrivial power–law exponents, size measures

## Abstract

Although the sizes of business firms have been a subject of intensive research, the definition of a “size” of a firm remains unclear. In this study, we empirically characterize in detail the scaling relations between size measures of business firms, analyzing them based on allometric scaling. Using a large dataset of Japanese firms that tracked approximately one million firms annually for two decades (1994–2015), we examined up to the trivariate relations between corporate size measures: annual sales, capital stock, total assets, and numbers of employees and trading partners. The data were examined using a multivariate generalization of a previously proposed method for analyzing bivariate scalings. We found that relations between measures other than the capital stock are marked by allometric scaling relations. Power–law exponents for scalings and distributions of multiple firm size measures were mostly robust throughout the years but had fluctuations that appeared to correlate with national economic conditions. We established theoretical relations between the exponents. We expect these results to allow direct estimation of the effects of using alternative size measures of business firms in regression analyses, to facilitate the modeling of firms, and to enhance the current theoretical understanding of complex systems.

## 1. Introduction

An index of the size of a business firm has numerous implications, ranging from its method of corporate finance [1,2] and the quality of its CEO [3] to employee job satisfaction [4] and gender gaps in wages among employees [5]. The exact determinants of the size of a specific firm or a group of firms in an industry have been sought both empirically [6,7] and theoretically [8,9]. Researchers have also studied regularities in the distribution of firm size empirically [10,11,12,13,14,15], mainly motivated by the theory that the evolution of firm size can be modelled as a stochastic process [10,16,17,18].

However, the question of what constitutes the “size” of a firm has rarely been addressed, and it is possible that statistical results regarding firm size depend on the measures used. Indeed, many of the effects previously found in empirical studies of corporate finance were not robust when different size measures were used as a control variable [19]. Researchers, who questioned the often implicit assumption that size measurements of firms are adequate indicators of their actual size [19,20,21], evidently agree that the relations of various size measures commonly used have not been well-explored in the literature. Moreover, some [20,21] propose the notion of multidimensional size for business firms, using various size measures to indicate manifold aspects. Although it might clarify the conceptual status of firm size, differences and relations between firm size measures should be explored empirically for the notion of multidimensional size to be fruitful.

Regarding the relations between corporate size measures, power-law scaling relations have been reported empirically [12,19,22,23,24]. More interestingly, some size measures have been reported to demonstrate allometric scaling [23], which means that the distribution of relative deviations from the average relation between two size measures was invariant regardless of firm size. In other words, when the logarithms of a size measure were regressed against the logarithms of another measure, the residuals were found to be surprisingly homoscedastic and independent of the “explanatory” variable; note that the use of formulae for regression analysis is solely intended for the characterization of multivariate joint distributions and does not imply causal relations between the size measures. This suggests that we may be able to employ the residual as a variable indicating the adjusted “proportion” between size measures of a firm, allowing us to isolate the effects of using alternative size measures. Nevertheless, the size measures considered in previous studies are not intended to represent all possibilities. It is unknown as to what extent allometric scaling accurately describes empirical relations regarding other size measures. Theories regarding relations between multiple allometric scalings have been lacking as well. Therefore, empirical characterization and theoretical understanding of various size measures are still far from complete.

Analysis of the empirical relations between size measures of firms also has merit for advancing complex systems science, and a few studies [23,24] have indeed been pursued from such a perspective. Power laws of the form $x\propto y^{\gamma }$ are a typical functional form of scaling found in general complex systems, such as animal bodies [25,26,27,28], ecological communities [29,30], and cities [31,32]. For instance, the value of exponent *γ* in the scaling of the metabolic rate (*x*) on the body mass (*y*) is close to 3/4 in mammals [25,27,28] and is theoretically related to the minimization of the energy consumption in blood pumping [33]. Theoretical considerations have successfully been used to predict numerous other scaling exponents in the natural systems of animal bodies. Similarly, the unique value of *γ* = 2 for humans (compared with *γ* = 3 for other animals) in the scaling of the body mass (*x*) against the body length (*y*) was theoretically accounted for by human bipedalism [34]. In this manner, studies on scaling relations provide a basis for a deeper understanding of a system’s governing principles.

The present study is focused on multivariate relations among five quantities of firms that have often been used as size measures in research and practice to characterize scaling relations exhibited by firms across scales. We based our study on exhaustive annual data for more than 2 million unique Japanese firms over a 22-year period. After examining the properties of individual size measures, we empirically verified statistical models of up to three-dimensional allometric scaling for four of the five size measures, while establishing theoretical relations between multiple scalings. Building upon these results, we further developed a method for estimating scaling exponent *γ* as well as the range where the data fit well to the scaling relations, thereby obtaining insights into the temporal robustness of our results.

The remainder of this paper is organized as follows. First, we describe our selection of firm size measures and the employed data in Section 2. After the dataset and statistical properties of each size measure are characterized in Section 3.1, we inspect the bivariate scalings between the size measures and specify the pairs of variables that are in an allometric scaling in Section 3.2. We then address the problems of seeming inconsistency between the power–law exponents for the tails of marginal and conditional distributions of size measures with regard to bivariate scalings (Section 3.3). Next, we examine a trivariate scaling relation in Section 3.4. Trivariate allometric scalings were found for some combinations of size measures and shown to be related to the transitivity of allometric scalings both theoretically and empirically. This theoretical development allows robust estimation of bivariate and trivariate scaling exponents (Section 3.5). We found that the number of employees has a higher relevance to sales than does the number of trading partners, and there was a mild variation in the trivariate scaling exponents in the period 1994–2015, which appeared to be correlated to the gross domestic product (GDP) variation (Section 3.6). Finally, we discuss our results and conclude the paper in Section 4. Our rationales for using the standard regression analysis are given in Appendix A, and problems related to the theoretical relations between several power–law exponents that appear in scalings, conditional distributions, and marginal distributions are discussed in detail in Appendix B.

## 2. Materials and Methods

### 2.1. Selection of Firm Size Measures

Despite the recurrent claim that the concept of firm size has not been sufficiently clarified [19,20,21], the variety of size measures employed in studies has remained mostly unchanged during the past half century. For example, in an economic study in 1975, the authors compared different firm size measures using the data of sales, total or net assets, employment, invested capital, and market value [22]. Almost contemporarily, a review of organizational studies in 1976 listed the number of employees as well as the number of clients, sales, and net assets as commonly used size measures and proposed physical capacities, such as the square footage (area) available for an organization’s activities, as a possible measure [20]. Recent research on corporate finance mentions total assets, sales, market value, and the number of employees [19]. Meanwhile, the econophysics school of research has used sales, number of employees, total assets, net sales, net income, and number of trading partners (i.e., the reported number of firms that a firm directly trades with) [12,23,35]. Finally, as small and medium enterprises are legally defined according to their capital stock in Japan [36], capital stock data are widely available for Japanese firms, which is the subject of the database used in our study.

We considered only the firm size measures that did not take negative values and were readily measured for almost all firms. The former criterion concerns the very notion of size in broader contexts. For instance, when the size of an animal body is considered, it is non-negative whether it is measured by weight, length, or how many calories per day it takes for the organism to live. We excluded net assets, net sales, and net income based on this criterion, as these measures can take negative values. Other implications of the concept of a size measure include positive correlations to other size measures—e.g., the weight of a human body is usually positively correlated to the height—but this was not incorporated into our criteria due to the empirical nature of the question. We set the latter criterion pragmatically, allowing us to preserve the comprehensiveness of our original data and to minimize the sampling bias. Market value was rejected with this criterion because data were unavailable for firms that were not publicly traded, which comprised the vast majority by number. Thus, the measures of corporate size that were referred to in the literature and that met our criteria were sales, total assets, invested capital, capital stock, and the numbers of employees and trading partners. We omitted invested capital from our analysis because of its partial conceptual overlap with capital stock, relative scarcity of the data, and its rare appearance in the literature as a size measure.

### 2.2. Data and Preprocessing

The source of the data used in this study was a collection of brief descriptions of Japanese business firms, including the financial status, type of business, and physical location, and this was provided and maintained by a major Japanese private credit reporter, Teikoku Databank, Ltd., Japan (hereinafter referred to as TDB). The data were mostly based on annual questionnaires presented to Japanese business firms.

The data employed in this study were primarily from the electronic version of the COSMOS 2 database, which is updated every January by TDB. This database describes over two million business firms in Japan and includes two sub-databases: (1) “summaries” of firm profiles, including the sales, capital stock, number of employees, and business type category (approximately one million per year, from 1980) and (2) lists of several million trading relationships between firms for each year since 1993. The profile of the trading relationships included the direction of money flows, i.e., which firm (buyer) paid which (supplier). Our study was focused on the data after 1993, in which the COSMOS 2 database started to capture the trading relations between firms. We excluded the 1993 data to avoid boundary effects that may have been present at the beginning of the data collection. The data for sales, number of employees, and trading relations that we employed were last updated in January 2017, whereas the data for the capital stock were compiled in January 2020. This confined our analysis to the period ending in 2015 because the 2016 sales were not available for a substantial portion of the firms. Data for the total assets were from the COSMOS 1 database by TDB, in which financial statements of firms were included. This database was last updated in May 2020, and data since 2000 were available to us. Each firm has a unique anonymous ID that allows the firm to be identified in different databases.

To ensure time coherence between different types of data and to make the included firms sufficiently homogeneous for the purpose of our analyses, we performed several data-compilation steps, as follows. First, we excluded the firms that were categorized as governmental (e.g., local governments) or financial (e.g., banks and insurers). This is because the definition of sales for these “firms” differed significantly from that for construction, manufacturing, and wholesale firms, which constituted the majority of the firms in the data. In this step, we filtered out possible outliers in the database. This procedure was applied to both databases. Second, we did not use sales and capital stock data from financial statements published more than 8 years before data entry or without an adequate timestamp. Third, we set the sales to be unknown when the end of the fiscal year had changed because some such data were apparently not the annual sales (the value was sometimes considerably lower than those in previous or subsequent periods), whereas we accepted the data of capital stock and total assets in such cases if more than one instance of inconsistent data did not appear for the same year. Fourth, we determined the year to which a datum of sales, capital stock, or total assets was assigned according to the year in which the fiscal term ended. For example, when a firm had a fiscal year that started in April 2000 and ended in March 2001 (as for the majority of Japanese firms), the sales value of the fiscal term was considered to belong to the year 2001, regardless of whether the data appeared in the database in 2002 or later. In contrast, this principle was not applied to the employee or trading data; we always assigned them to the year prior to the data entry, because the COSMOS 2 database was updated in January of each year.

We directly used the raw data without normalization or adjustment for inflation. The number of trading partners of a firm was determined by counting the trading relations in which the firm participated throughout the year, regardless of whether the firm was a supplier or a buyer. This amounted to computing the sum of the in-degree and out-degree for each node in the directed network of trading.

The data for firm exits were additionally compiled to estimate the number of firm entries and exits. First, if any data of a firm existed in the COSMOS 2 database for a year, the firm was considered to exist in that year. Additionally, when the data of a firm were unavailable for up to two consecutive years, the firm was considered to exist in these years, assuming that its absence from the data was accidental. Before the compilation of the exit data, the first and fourth steps of data exclusion described earlier for the compilation of quantitative data were applied to ensure consistency between the datasets.

When multiple variables were used in the analyses, we excluded the data of firms that lacked any one of the variables. Results for the data amount are presented in Section 3.1 and tabulated in Table S1.

## 3. Results

### 3.1. Characterization of Databases and Size Measures

**Figure 1:**
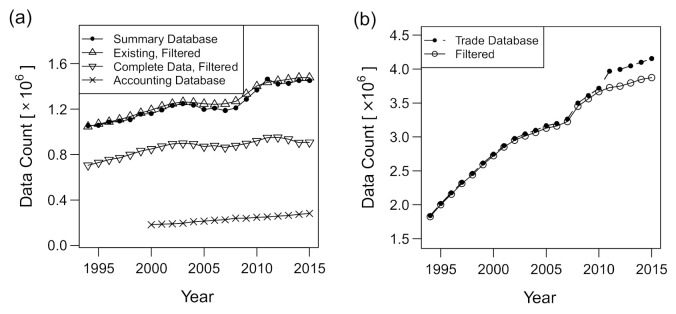

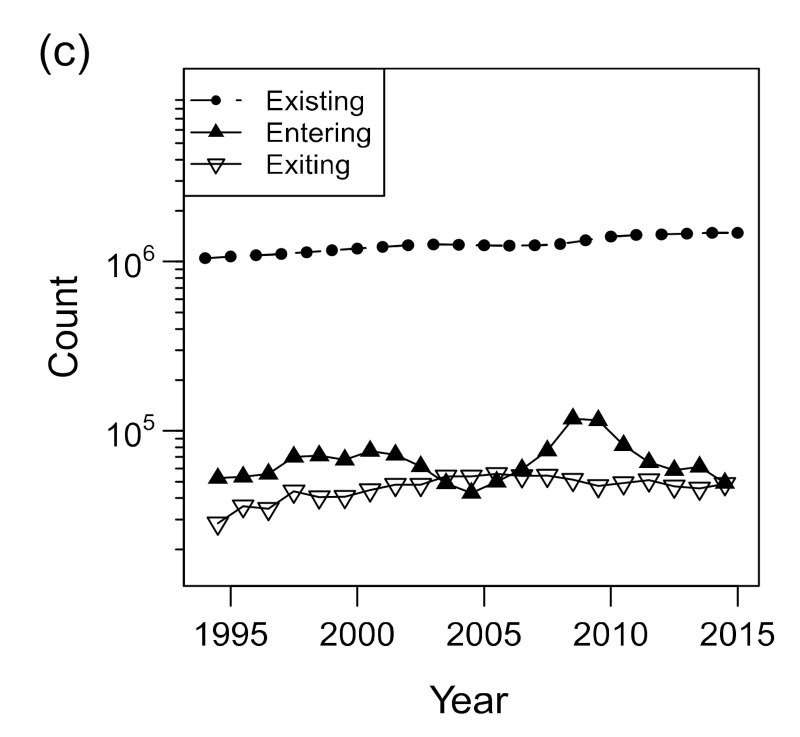
Changes in the data amount for the period 1994–2015. (**a**) Data amount for the original COSMOS 2 database (black dots), number of existing firms after the filtering and integration with network data (upward triangles), number of firms with data of all the three variables of sales, number of employees, and number of trading partners equal to or larger than one (downward triangles), and number of firms with total asset information in the COSMOS 1 database after the filtering (cross marks). (**b**) Data amount (number of links) for the original trade database (dots) and after the filtering (circles). (**c**) Numbers of entering (upward triangles) and exiting (downward triangles) firms. The number of existing firms (dots) is plotted for comparison. The vertical axis is in the logarithmic scale.

The amounts of data before and after the compilation are plotted in Figure 1 with respect to the years in the 1994–2015 period. The amount of data generally increased. The number of firms with complete data for multiple size measures in the COSMOS 2 database (i.e., number of trading partners *k*, number of employees *ℓ*, and annual sales *s*) was more than 0.8 million per year in 1999 and later, and the data for total assets *a* were more than 0.2 million per year in 2004 and later, as shown in Figure 1a, after the filtering procedure described in Section 2.2. The number of firms considered to be existing based on the COSMOS 2 database was usually larger than the number of firms in the sub-database of sales and employees, as existence was assumed for some firms that only appeared in the trading data or those with temporarily missing data. Two sudden increases in the number of trading data (2007–2008 and 2010–2011) are observed in Figure 1b. The second jump was due to trading data involving financial or governmental organizations, as we observed no jump in the same period after the filtering. In contrast, the first jump was the consequence of revised data-collection methods that were specific to trading-relations data; trading relationships that had been mentioned only in on-demand reports started to be included in the database in 2008. Indeed, there was no comparable jump in the number of “summary” profile data. Additionally, the number of exiting or disappearing firms was stable compared with the number of entering firms, as shown in Figure 1c, implying that the former was less affected by the fluctuation of the data-collection efforts by TDB.

**Figure 2:**
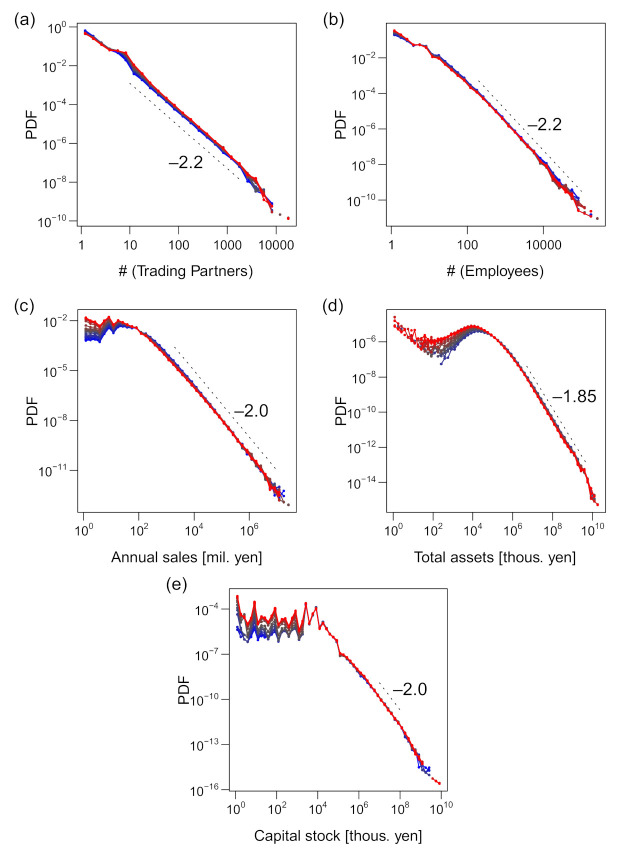
Probability distribution function (PDF) of the size variables for the period 1994–2015. The figures are plotted on log–log scales. Numerals near the dashed lines indicate the slope of the lines. The blue-to-red gradient of the color indicates the direction from old to new data. (**a**) Number of trading partners. (**b**) Number of employees. (**c**) Annual sales in millions of yen. (**d**) Total assets in thousands of yen. (**e**) Capital stock in thousands of yen. Data of total assets are plotted for the year 2000 or after, where the data were available.

All the size variables were distributed in fat-tailed manners on the right side, as shown in Figure 2. Their distribution functions for every year are plotted in the figure, with the color gradient from blue through black to red, indicating the direction from older data to newer ones. The distributions were fairly stable despite the increase in data and, in particular, the exponents of the power–law tails were evidently invariant when the tails could be approximated by a power–law distribution. They were –2.2 for *k* (the number of trading partners), –2.2 for *ℓ* (employee number), –2.0 for *s* (annual sales in million yen), and –1.85 for *a* (total assets in thousand yen). The exponent of the sales distribution was consistent with that in previous studies [13,14,15]. The distribution of capital stock (hereafter denoted by *p*) was also fairly fat-tailed, although it did not seem to be characterized by a power law because the curve of the estimated density function was convex, as seen in Figure 2e. Consequently, the arithmetic mean of *s* and *a* did not indicate the representative value, and the standard deviation of all the variables was not a robust index of the width of the distribution (Table S1). We alternatively indicated the representative value and width of distribution by the median and the interquartile range, respectively, as shown in the table. However, these values were only representative of firms of middle-range sizes, not the ones at the tails, which were characterized by power–law distributions and scaling relations.

The capital stock was unique because the right tail did not exhibit a power law and there were multiple modes near the median value. A manual inspection of the data revealed that these multiple modes were the result of preference for rounded values such as 10,000. This phenomenon was also notable in other published data [36].

### 3.2. Bivariate Scalings between Size Measures

The dependencies between the size measures of business firms were characterized in terms of bivariate “allometric” scaling relations in a previous study [23], and we visualized them using the method described in the study. The data in the previous study were gathered by a credit reporting company independent of TDB. However, we expected the results to be similar between the two datasets, as the data were collected for the same system (Japanese firms).

Bivariate allometric scaling is denoted by $x\propto y^{\gamma }$, where *γ* represents the exponent, and *x* and *y* are two different size measures. We first consider the three size measures studied previously: the number of trading partners (*k*), number of employees (*ℓ*), and annual sales in millions of yen (*s*). As described in the previous study [23], the concept of allometric scaling relations incorporates scale-invariant relative fluctuations and is defined in terms of conditional distributions:

(1) $$P\left(l|k\right)\;={\tilde{P}}_{l|k}\left(l/k^{\gamma_{1}}\right)/k^{\gamma_{1}},$$

(2) $$P\left(s|k\right)\;={\tilde{P}}_{s|k}\left(s/k^{\gamma_{2}}\right)/k^{\gamma_{2}},$$

(3) $$P\left(s|l\right)\;={\tilde{P}}_{s|l}\left(s/l^{\gamma_{3}}\right)/l^{\gamma_{3}},$$

where $P\left(x|y\right)$ represents the probability density function of variable *x* conditional on a specific *y*-value; $\gamma_{1}$, $\gamma_{2}$, and $\gamma_{3}$ represent the scaling exponents; ${\tilde{P}}_{x|y}$ represents a scaling function for variable *x* conditional on the *y*-value, which indicates the distribution of the fluctuation ratio around the scaling line, $x\propto y^{\gamma }$.

All the probability distribution functions of the three size variables (*k*, *ℓ*, and *s*) were fat-tailed for large values in each year (see Figure 1a–c and Table S1). To avoid extreme values, which are usually observed in variables distributed in such a manner, we log-transformed the raw size data so that the variables were exponentially distributed. After the log-transformation, Equations (1)–(3) were more intuitive linear regression-type formulae, as discussed in detail in Appendix A:

(4) $$\log l=\gamma_{1}\log k+\varepsilon_{l|k},$$

(5) $$\log s=\gamma_{2}\log k+\varepsilon_{s|k},$$

(6) $$\log s=\gamma_{3}\log l+\varepsilon_{s|l}.$$

**Figure 3:**
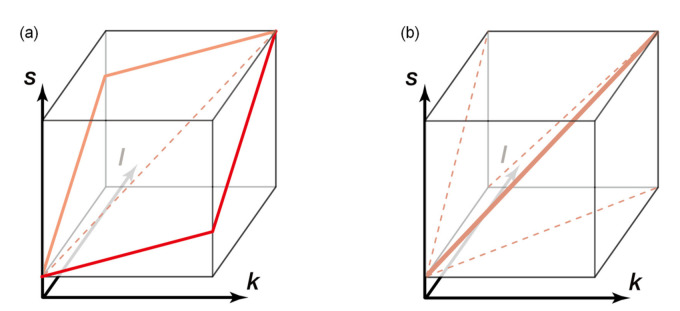
Schematics of the scaling relations. A firm is represented as a point in the three-dimensional phase space. Variables *k*, *ℓ*, and *s* represent the number of trading partners, number of employees, and annual sales, respectively. (**a**) Multivariate scaling *s*∝*k^α^ℓ^β^* is illustrated as a plane (red solid line). (**b**) Three bivariate scaling relations (indicated by the dashed lines) can be understood as projections of a single (red bold) “scaling line.” The plane in panel (**a**) must include the scaling line (red dashed), but this line clearly cannot determine a unique plane.

Here, the error terms, $\varepsilon_{l|k}$, $\varepsilon_{s|k}$, and $\varepsilon_{s|l}$, can be regarded as stochastic variables that correspond to ${\tilde{P}}_{l|k}$, ${\tilde{P}}_{s|k}$, and ${\tilde{P}}_{s|l}$, respectively. The mean of these “error terms” is generally not equal to zero, and the intercept term (as it is called in regression analysis) is included among them. These bivariate scaling laws, presented in a previous work [23], can be naturally interpreted as projections of a single three-dimensional scaling line onto two-dimensional planes, as illustrated in Figure 3a, around which the firms are densely distributed [37]. A similar concept of multivariate scaling was used in a study on the morphology of organisms [38].

To test the validity of these relations (Equations (1)–(6)) in our data, we examined both the conditional quantiles and the distributions of the error terms, where the conditional quantile was defined as follows: for any given number, *q,* in the range of (0, 1), the *q*-quantile of *x* conditional on *y*, which is denoted as $\langle x|y\rangle_{q}$, satisfies the following equation:

(7) $$\int_{0}^{\langle x|y\rangle_{q}}P\left(x|y\right)dx=q.$$

If Equations (1–6) hold, all the quantiles of the same *q*-value conform to the power–law relation, $\langle x|y\rangle_{q}\propto y^{\gamma },$ and the curves of $\langle x|y\rangle_{q}$ plotted against *y* for different *q*-values should collapse into a single curve when vertically shifted. Furthermore, the distributions of $x/\langle x|y\rangle_{0.5}$ conditional on *y* should be identical for all *y*-values and amount to the distribution specified by ${\tilde{P}}_{x|y}$ with normalization by its median.

**Figure 4:**
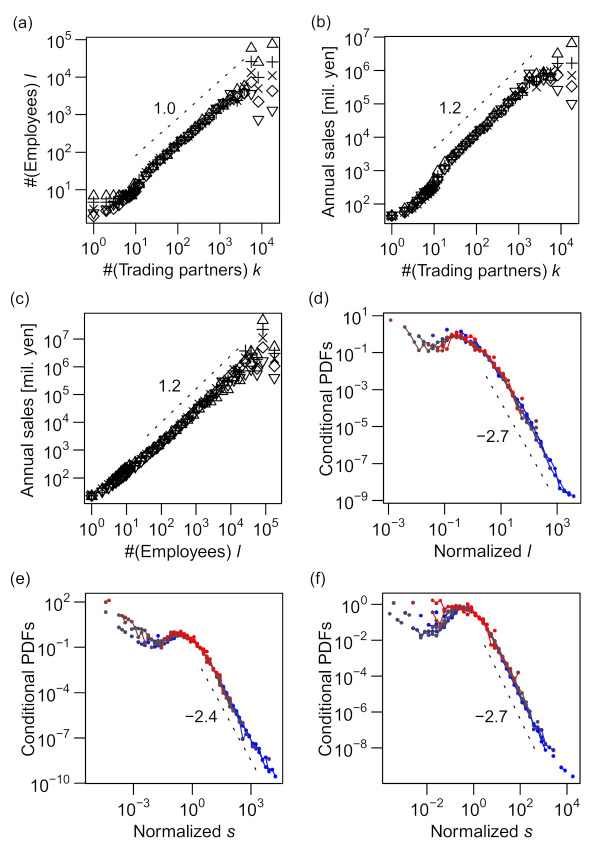
Scaling relations between pairs of measures: number of trading partners *k*, number of employees *ℓ*, and annual sales in millions of yen *s*. All the data used in the figures are for 2014. (**a**–**c**) 0.95(▽), 0.75(+), 0.5(×), 0.25(◇), and 0.05(△) quantiles of the conditional distribution of *ℓ* or *s* (vertical axis) are plotted against another variable *k* or *ℓ* (horizontal axis) on a log–log scale. The horizontal axis is divided into intervals of identical lengths on the log scale (6 segments per a 10-fold interval such as 10–100) for *k* and *ℓ* values of >10 and intervals of length unity on the linear scale for *k* and *ℓ* values of ≤10. Quantiles other than those of *q* = 0.5 (i.e., medians) are plotted with horizontal shift, so that all the curves pass through a point whose *x*-axis is slightly above 100. (**d**–**f**) Probability distributions (PDFs) of *ℓ* or *s* conditional on *k* or *ℓ* (i.e., $P\left(l|k\right)$, $P\left(s|k\right)$, and $P\left(s|l\right)$ ) for 8 different “bins” (i.e., mutually exclusive intervals) of the “explanatory” variable (*k* or *ℓ*), normalized by their conditional medians, plotted on a log–log scale. The bins are obtained by evenly dividing the entire range (from the minimal value of unity to the maximum value of the variable shown in Table S1) into eight segments on the logarithmic scale. The color gradient of blue to red indicates low to high values of the explanatory variable.

As shown in Figure 4a–c, the plots of $\log\;\langle x|y\rangle_{q}$ vs log y indicated linear relations for a wide range of *y*, indicating that the power–law relation, $\langle x|y\rangle_{q}\propto y^{\gamma }$ (abbreviated as $x\propto y^{\gamma }$), holds for all combinations of *k*, *ℓ*, and *s*. In Figure 4d–f, the distribution of relative fluctuation from the conditional median ($x/\langle x|y\rangle_{0.5}$) is plotted for eight intervals of equal length on the logarithmic scale of *y*. All the curves collapse into a single “scaling function” for all three cases, validating the scaling relations of Equations (1)–(3).

**Figure 5:**
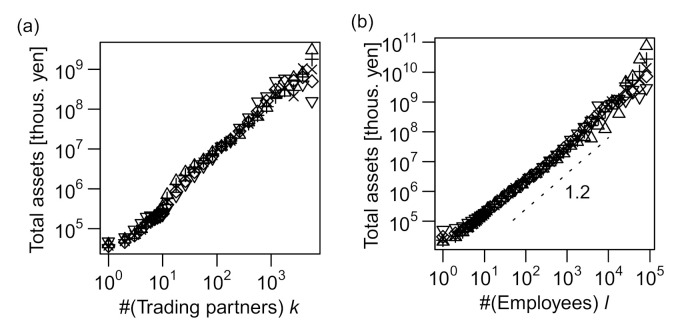

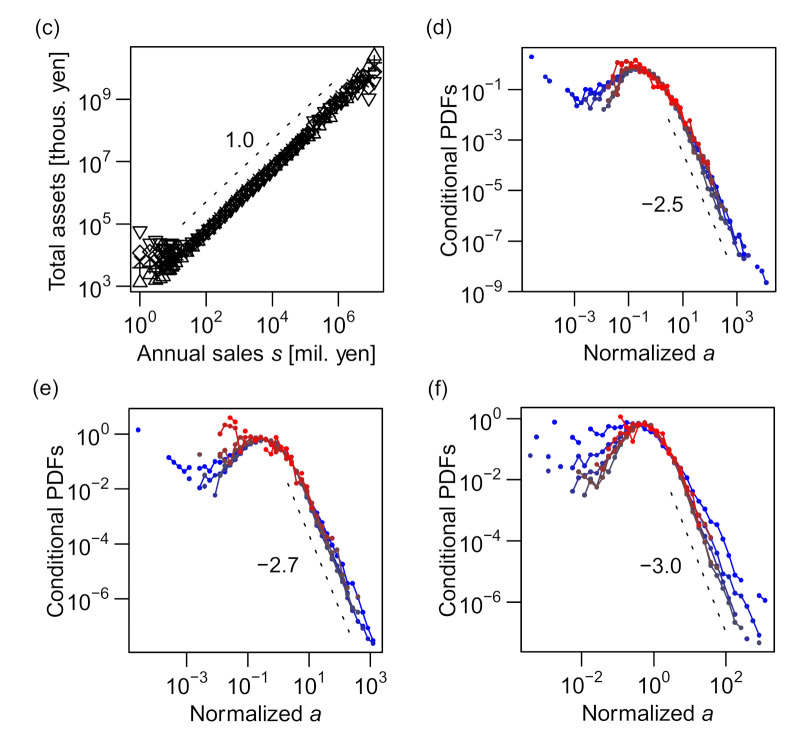
Scaling relations between total assets in thousands of yen *a* and number of trading partners *k*, number of employees *ℓ*, and annual sales in millions of yen *s*. All the data used in the figures are for 2014. (**a**–**c**) Vertically shifted quantiles of the conditional distribution of *a* (vertical axis) are plotted against another variable *k*, *ℓ*, or *s* (horizontal axis) on a log–log scale. (**d**–**f**) Probability distributions (PDFs) of *a* conditional on *k*, *ℓ*, or *s* (i.e., $P\left(a|k\right)$, $P\left(a|\ell \right)$, and $P\left(a|s\right),$ respectively) for 8 different “bins” of the “explanatory” variable (*k*, *ℓ*, or *s*), normalized by their conditional medians, plotted on a log–log scale. See the caption of Figure 4 for details on plotting.

We next examined the total assets, *a*, with regard to *k*, *ℓ*, and *s*. The relation between *k* and the quantiles of *a* ($\langle a|k\rangle_{q}$) was not clearly linear, being slightly curved in a logarithmic plot, as shown in Figure 5a; however, the distributions of relative fluctuations from the conditional median ($a/\langle a|k\rangle_{0.5}$) were almost independent of *k* (Figure 5d). More obvious allometric scaling can be seen for *ℓ*–*a* (Figure 5b,e) and *s*–*a* (Figure 5c,f) relations. The total assets seemed to scale superlinearly with *ℓ* (*γ* > 1) and linearly with *s* (*γ* = 1). The power–law scaling exponents of *a* with regard to *ℓ* and *s* are hereafter denoted by *γ*_4_ and *γ*_5_, respectively. Note that the distributions of relative fluctuations of *a* (i.e., $a/\langle a|s\rangle_{0.5}$) were wider for small values of *s* (blue curves) than for large values (red curves), as seen in Figure 5f. We also noted an increase in *a* slightly larger than a linear function of *s* for sales values more than 10^5^. Therefore, allometric scaling most precisely approximated the *s*–*a* relation for the range of *s* between 10^2^ and 10^5^. We conclude that total assets scaled allometrically with *ℓ* and *s*, and the relation between *a* and *k* did not conform to the allometric scaling.

**Figure 6:**
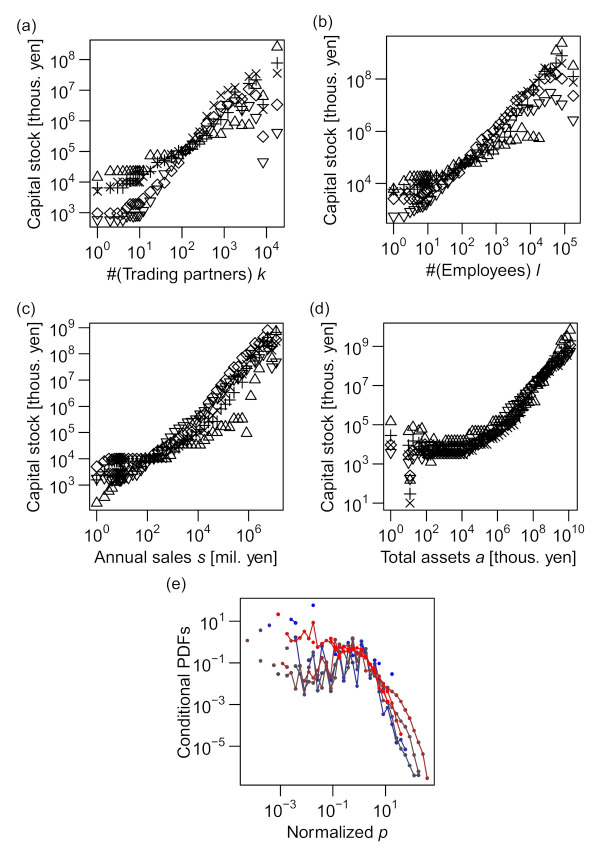
Scaling relations between capital stock and number of trading partners *k*, number of employees *ℓ*, annual sales in millions of yen *s*, and total assets in thousands of yen *a*. All the data used in the figures are for 2014. (**a**–**d**) Vertically shifted quantiles of the conditional distribution of *p* (vertical axis) are plotted against another variable *k*, *ℓ*, *s*, or *a* (horizontal axis) on a log–log scale. (**e**) Probability distributions (PDFs) of *p* conditional on *a* (i.e., $P\left(p|a\right)$) for 8 different “bins” of the “explanatory” variable (*a*), normalized by their conditional medians, plotted on a log–log scale. See the caption of Figure 4 for details on plotting.

The capital stock, *p*, was not found to be in an allometric scaling with any of *k*, *ℓ*, *s*, or *a*, as shown in Figure 6. The vertically shifted curves of conditional quantiles did not agree for *k*–*p*, *ℓ*–*p*, and *s*–*p* relations (see Figure 6a–c), indicating that the forms of conditional distributions of *p* differed substantially as the values of *k*, *ℓ*, or *s* varied. Whereas the *a*–*p* relation appeared to be close to allometric in the plot of conditional quantiles, as shown in Figure 6d, the conditional relative fluctuations ($p/\langle p|a\rangle_{0.5}$) were not constant over the range of *a* (Figure 6e).

### 3.3. Explaining Puzzling Scaling Exponents

Next, we studied the open question of the different power–law exponents in the conditional and marginal sales distributions, which has not been addressed previously. This is related to the asymmetric levels of relevance of the numbers of employees (*ℓ*) and trading partners (*k*) to the firm sales (*s*), as described in the following section.

**Figure 7:**
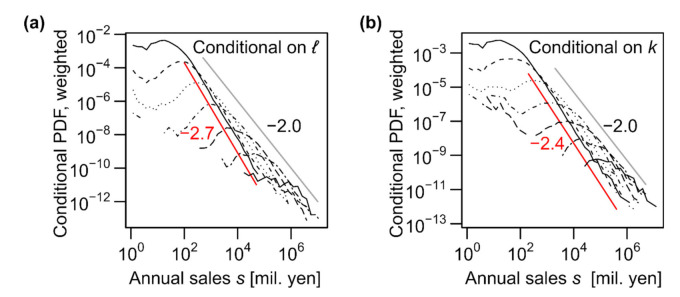
Apparent inconsistency of the power–law tail exponents for marginal and conditional PDFs, with an intuitive understanding based on Bayes’ theorem. Conditional probability distributions (**a**) P(*s*|*ℓ*) and (**b**) P(*s*|*k*), weighted with P(*ℓ*) and P(*k*) (see Equation (12)), respectively, plotted on log–log scales. The entire range of *k* and *ℓ* is divided into eight levels corresponding to eight curves, so that each interval has an identical range on the logarithmic scale. The weight of an interval is defined as the average probability density in the interval. All the data used in the figures are for 2014.

It is well established that the distribution of annual sales *s* of firms roughly follows Zipf's law [13,14,15]; i.e., the probability density tail follows a power law, P(*s*) ∝ *s*^–2^. This was also observed in our data (see Figure 2c). However, for the conditional sales distributions, the power–law exponents increased to approximately 2.4 (for the number of trading partners (*k*); see Figure 4e) and approximately 2.7 (for the number of employees (*ℓ*); see Figure 4f), substantially varying from 2.0 (see Figure 7).

These seemingly contradictory results are explained by Bayes’ theorem. Considering number of employees *ℓ* and annual sales *s*, we can approximate the integral of $P\left(s|\ell \right)P\left(\ell \right)$ with respect to *ℓ* using the maximum values of $P\left(s|\ell \right)P\left(\ell \right)$:

(8) $$P\left(s\right)\;=\int_{\ell }P\left(s|\ell \right)P\left(\ell \right)d\ell \;\sim \;P\left(s|\ell_{\mathrm{lead}}\right)P\left(\ell_{\mathrm{lead}}\right)\Delta \ell_{\mathrm{lead}},$$

where $\ell_{\mathrm{lead}}=\arg\max_{\ell }P\left(s|\ell \right)P\left(\ell \right)$, such that $P\left(s|\ell_{\mathrm{lead}}\right)P\left(\ell_{\mathrm{lead}}\right)$ is the “leading order” contribution, and Δ*ℓ*_lead_ is the width of *ℓ* at *ℓ*_lead_, which is assumed to be a constant. In Figure 5a, the functional form of $P\left(s|\ell \right)P\left(\ell \right)$ is plotted for several typical values of *ℓ* based on real data. As shown, the envelope function of $P\left(s|\ell \right)P\left(\ell \right)$ followed a power law with an exponent close to –2.0 at its tail. Similar results were obtained for the number of trading partners (*k*), as shown in Figure 5b. A more rigorous derivation is presented in Appendix B.

Because the scaling relations between the size variables, i.e., *ℓ*∝*k*^1.0^, *s*∝*k*^1.2^, and *s*∝*ℓ* ^1.2^ (see the following sections for the determination of these exponents), suggest symmetric relevance of *k* and ℓ to annual sales *s*, it is surprising that different exponents of the power–law tails for different conditional distributions (shown in Figure 5a,b) suggest strong asymmetry between the levels of relevance of *k* and *ℓ* to *s*. We discuss this novel feature in more detail in the next section in connection with a trivariate scaling relation.

### 3.4. Multivariate Scaling with Scale-Invariant Fluctuation

So far, we have studied bivariate relations between size measures of business firms. In spite of the intuitive picture of triple bivariate scalings as the two-dimensional projections of a single three-dimensional scaling line (Figure 3b), the transitivity of allometric scalings is not theoretically guaranteed; for example, $\ell \propto k^{\gamma_{1}}$ and $s\propto \ell^{\gamma_{3}}$ does not necessarily mean $k\propto s^{\gamma_{1}\gamma_{3}}$. Although the transitivity might be justified if the distribution of *s* is constant for an *ℓ*-value regardless of the *k*-value, such an assumption directly contradicts the previous results [23]. Therefore, trivariate relations should be considered to understand the theoretical basis of the transitivity of allometric scaling relations in the empirical datasets.

We first inspected the scaling of annual sales *s* with regard to the numbers of trading partners and employees, *k* and *ℓ*. Our analysis assesses the relative relevance of the number of trading partners (*k*) and the number of employees (*ℓ*) to the prediction of annual sales *s*. We generalize the allometric scaling relations to a multivariate relation, as follows:

(9) $$\log s=\alpha \log k+\beta \log\ell +\varepsilon_{s|k,\ell }\;$$

or

(10) $$P\left(s|k,\ell \right)={\tilde{P}}_{s|k,\ell }\left(s/k^{\alpha }\ell^{\beta }\right)/k^{\alpha }\ell^{\beta },$$

where *α* and *β* are the scaling exponents indicating the relative relevance of *k* and *ℓ*, respectively; *ε_s|k,ℓ_* is a stochastic fluctuation term of log *s* conditional on both *k* and *ℓ*; $P\left(s|k,\ell \right)$ represents the conditional probability density, which is dependent on both *k* and *ℓ*; ${\tilde{P}}_{s|k,\ell }$ represents the scaling function. This multivariate scaling relation was proposed in [23] but not confirmed directly by real data. Assuming that Equation (7) is satisfied, the median value can be straightforwardly derived:

(11) $$\log\;\langle s|k,\ell \rangle_{0.5}=\alpha \log k+\beta \log\ell +\langle \varepsilon_{s|k,\ell }\rangle_{0.5},$$

where $\langle s|k,\ell \rangle_{0.5}$ represents the median value of *s* conditional on a specific set of *k* and *ℓ* values. If Equation (9) holds, the contour plots of conditional median sales $\langle s|k,\ell \rangle_{0.5}$ on the *k*-*ℓ* logarithmic coordinate plane should exhibit nearly regular and parallel contours. Because $\langle \varepsilon_{s|k,\ell }\rangle_{0.5}$ in Equation (9) is a constant, the multivariate scaling relation can be represented by a plane, as shown in Figure 3a. Importantly, this differs from the “scaling line” [37], which is implied by the three scaling relations for the pairs of variables (Figure 3b).

**Figure 8:**
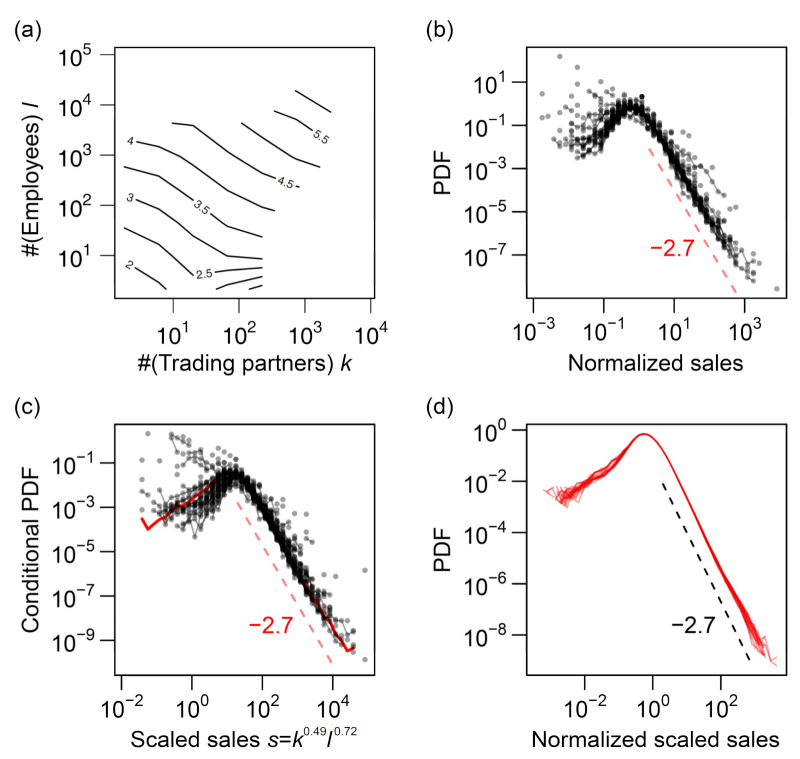
Multivariate scaling relations of annual sales *s* against number of trading partners *k* and number of employees *ℓ*. The plots in all the panels are on a log–log scale and present results for the 2014 data, unless otherwise mentioned. (**a**) Contour plot of the median values of annual sales *s* in millions of yen, conditional on both the number of trading partners, *k* (horizontal), and the number of employees, *ℓ* (vertical). The entire ranges of *k* and *ℓ* are divided into eight levels, so that each interval has an identical range on the logarithmic scale. The contours are obtained by linearly interpolating the log-transformed median sales values. Data of the conditional median from a grid with less than 10 samples were omitted in the plot. (**b**) Probability distributions (PDFs) of *s* conditional on both *k* and *ℓ*, normalized by their medians. The conditional distributions are obtained for grids of the conditioning variables where both dimensions are divided into intervals of identical length on a log scale (2 segments per a 10-fold interval). (**c**) PDFs of scaled sales *s/kαℓβ* conditional on both *k* and *ℓ*, where *α* and *β* represent the estimated exponents. The binning method of *k* and *ℓ* is identical to that for panel (**b**). Red lines indicate the marginal probability distribution of *s/kαℓβ*. (**d**) Probability distributions of *s/kαℓβ* for each of the 22 years (1994–2015), normalized with respect to the medians. The exponents used are the estimated values for each year, as shown in Figure 10c.

Although the relation is not a perfect plane but a surface because it is curved in the high-*k* and low-*ℓ* regions, as shown in Figure 8a, the data support the scaling of conditional median sales against the *k* and *ℓ* values (Equation (9)), particularly for medium or large values. Moreover, the statistical fluctuations around the median value are clearly independent of *k* and *ℓ* (Figure 8b). These facts indicate that the assumption of scaling represented by Equation (8) is valid for most *k* and *ℓ* values. When the distribution of scaled sales *s*/*k^α^ℓ^β^* conditional on *k* and *ℓ* was plotted using the values of *α* and *β* estimated according to the data (Figure 8c), a remarkable fraction of the curves scaled with each other. Additionally, function ${\tilde{P}}_{s|k,\ell }$ was surprisingly stable across the years (Figure 8d). Thus, the scaling assumptions of Equations (7) and (8) are well supported by a large amount of available data.

The scaling exponent for *k* (*α*) was smaller than the scaling exponent for *ℓ* (*β*), as indicated by the moderate slopes of the contours compared with –1.0 in Figure 8a. We quantitatively verify this statement in the following section. This finding can be linked to the asymmetricity of *k* and *ℓ*, as discussed in the previous section. In view of the magnitude of errors or fluctuations around the scaling relations, the distribution of residuals (*ε_s|k_* and *ε_s|ℓ_*, as defined in Equations (5) and (6)) was more fat-tailed when *s* was regressed against *k* (${\tilde{P}}_{s|k}\left(\tilde{s}\right)\propto {\tilde{s}}^{-2.4}$; see Figure 4e) than when *s* was regressed against *ℓ* (${\tilde{P}}_{s|\ell }\left(\tilde{s}\right)\propto {\tilde{s}}^{-2.7}$; see Figure 4f). This difference in the tails of the error distributions implies that the number of trading partners (*k*) was less useful in predicting the sales value than the number of employees (*ℓ*), which was indeed the case.

**Figure 9:**
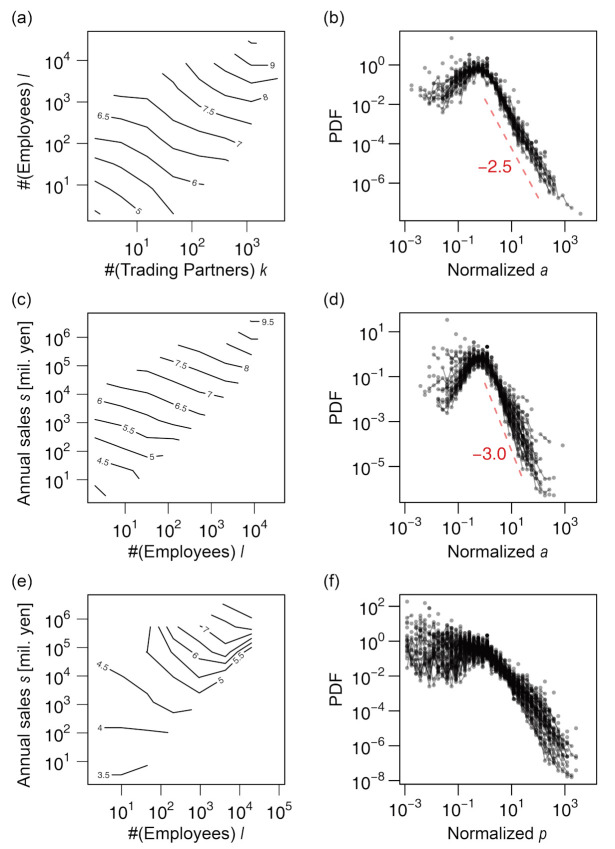
Multivariate scaling relations among number of trading partners *k*, number of employees *ℓ*, annual sales *s*, total assets *a*, and capital stock *p*. The plots in all the panels are on a log–log scale and present results for the 2014 data. (**a**) Contour plot of the median values of total assets *a* in thousands of yen, conditional on both the number of trading partners, *k* (horizontal), and the number of employees, *ℓ* (vertical). (**b**) Probability distributions (PDFs) of *a* conditional on both *k* and *ℓ*, normalized by their medians. (**c**,**e**) Contour plot of the median values of total assets *a* or capital stock *p* in thousands of yen, conditional on both the number of employees, *ℓ* (horizontal), and the annual sales, *s,* in millions of yen (vertical). (**d**,**f**) Probability distributions (PDFs) of *a* or *p* conditional on both *ℓ* and *s*, normalized by their medians. In contour plots, the entire ranges of the horizontal and vertical axes are divided into eight levels, so that each interval has an identical range on the logarithmic scale. See the caption of Figure 8 for further details on plotting.

The trivariate allometric scaling, *s*∝*k^α^ℓ^β^*, along with $\ell \propto k^{\gamma_{1}}$ and a power–law tail of the distribution of *k*, is sufficient to derive $s\propto \ell^{\gamma_{3}}$ and $k\propto s^{\gamma_{1}\gamma_{3}}$ asymptotically under fairly mild assumptions (see Appendix B). In other words, the transitivity of bivariate scaling relations depends on whether the trivariate allometric scaling holds among the variables. We compared the triplets of (*k*, *ℓ*, *a*) and (*ℓ*, *s*, *a*) to exemplify this point. Note that the transitivity held among the latter but not among the former, because allometric scaling holds between *k* and *ℓ* and between *ℓ* and *a*, but not between *k* and *a*. We expected that the tri-variate allometric scaling would hold only for the latter. The results for these combinations of variables that are analogous to Figure 8b are plotted in Figure 9a–d. Although the deviations of *a* from the conditional median were independent of *k* and *ℓ* (panel (b)), the contour curves representing the conditional median of *a* were notably curved against *k* and *ℓ* (panel (a)). In contrast, contours for *a* conditional on *ℓ* and *s* were straight, parallel to each other, and evenly spaced (panel (c)), and most of the deviations might be approximated by a single function (panel (d)). Therefore, the trivariate scaling of *a* with regard to *ℓ* and *s* can be approximated by the following equation:

(12) $$\log a=\alpha^{\prime }\log\ell +\beta^{\prime }\log s+\varepsilon_{a|\ell ,s}.$$

However, the trivariate scaling among *k*, *ℓ*, and *a* cannot be described in an analogous way as expected.

Finally, we inspected the trivariate scaling of the capital stock, *p*, against other size measures. The results for the three variables (*ℓ*, *s*, *p*) are shown in Figure 9e,f. Both figures indicate clearly that these three variables were not in an allometric scaling, as we can expect from the non-allometric bivariate scaling property of *p* discussed in Section 3.2.

### 3.5. Robust Estimation of Scaling Exponents

Using the trivariate scaling with a scale-invariant relative fluctuation, we can derive several mathematical relations between the scaling exponents (see Appendix B). Here, we show that these relations allow the asymptotic scaling exponents to be estimated robustly against scaling for small firms that does not conform to the power law.

By assuming the trivariate scaling formulated by Equation (7), bivariate scaling of a similar form between *k* and *ℓ*, and power–law tails of several conditional and marginal distributions, we can determine the joint probability distribution of *k*, *ℓ*, and *s*. Thus, one can obtain a set of relations between numerous scaling exponents. Central to these are

(13) $$\gamma_{2}=\alpha +\beta \gamma_{1},$$

and

(14) $$\gamma_{3}=\;\left(\alpha +\beta \gamma_{1}\right)/\gamma_{1},$$

where $\gamma_{1}$, $\gamma_{2}$, and $\gamma_{3}$ represent the *k*–*ℓ*, *k*–*s*, and *ℓ*–*s* scaling exponents, respectively (see Appendix B for the derivations). These theoretical equations are useful for estimating the exponents, as determining the threshold size over which the behaviors of firms can be approximated as asymptotic is critical to such an estimation. The aforementioned relations are satisfied only when the data with *k* < 100 or *ℓ* < 100 are excluded (see below), indicating that firms do not follow exactly the same power–law scaling below and above the threshold.

**Figure 10:**
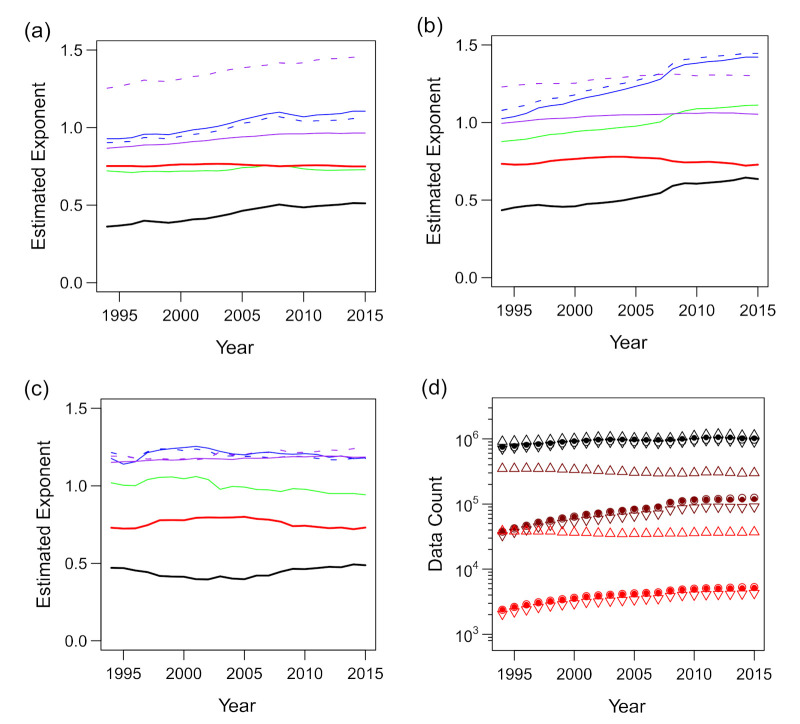
Estimated exponents of the trivariate scaling relations of *s* against *k* and *ℓ* for the period 1994–2015. Estimated exponents $\gamma_{1}$ in $\ell \propto k^{\gamma_{1}}$ (green), $\gamma_{2}$ in $s\propto k^{\gamma_{2}}$ (blue), $\gamma_{3}$ in $s\propto \ell^{\gamma_{3}}$ (purple), and α (black) and β (red) in $s\propto k^{\alpha }l^{\beta }$ are plotted. Additionally, the expected values of $\gamma_{2}$ and $\gamma_{3}$ derived mathematically from α, β, and $\gamma_{1}$ in the case of perfect scaling (Equations (10) and (11)) are juxtaposed (blue and purple dashed lines, respectively). (**a**) All the available data are used to estimate the exponents. (**b**) Only the data for which the “explanatory” variable is >10 are used. (**c**) Only the data for which the “explanatory” variable is >100 are used. (**d**) Plot of the size of samples from which the exponents are estimated against the year. The black, brown, and red points indicate the thresholds of 0 (no exclusion), 10, and 100, corresponding to panels (**a**–**c**), respectively. The hollow (○) and filled (●) circles and upward (△) and downward (▽) triangles represent the scaling of *ℓ* vs. *k*, *s* vs. *k*, *s* vs. *ℓ*, and *s* vs. *k* and *ℓ*, respectively.

We observed deviations from the scaling relations, particularly when the number of trading partners (*k*) and the number of employees (*ℓ*) were less than 10 or the annual sales (*s*) were less than 100 (Figure 4a–c, Figure 5a–c and Figure 8a; also see [37]). Because smaller firms dominated the data (Figure 2), their deviation from the scaling relations substantially affected the estimation of the scaling exponents. When all the available data were included in the regression analyses, there was a clear difference between the expected value of *γ*_3_ (dashed purple line) based on Equation (11) and the value obtained via direct estimation (solid purple line), as shown in Figure 10a.

We performed a standard regression analysis using R (ver. 3.1.2) to estimate the scaling exponents in the bivariate and multivariate scaling relations of the firm data. See Appendix A for the rationales for applying the regression analysis to the problem of estimating scaling exponents of power–law relations with scale-invariant relative fluctuations. To reject the data of small firms that did not fit the “linear” assumption of the model, we excluded the data with low values of “explanatory” variables in the regression formulae by applying a threshold. We used two values for possible thresholds: 10 and 100. The threshold was determined according to the consistency of the resulting multivariate scaling exponents with the bivariate ones. If the “explanatory” variables on the right-hand sides of Equations (4)–(7) were below the threshold, the datum was neglected. Although a considerable proportion of the data might be excluded from the analysis, this process ensured that the final set of exponents conformed to the model assumptions.

The results of regression of *s* against *k* and *ℓ* using thresholds of 10 and 100 are presented in Figure 10b,c, respectively. As shown, *γ*_1_ nearly reached 1.0, and the direct and indirect estimates of *γ*_3_ agreed only when the threshold was set as 100. This suggests that the threshold should be ≥100 for *k* and *ℓ*. We preferred to employ a smaller threshold value owing to the large sample size; thus, we adopted the value of 100. The sample sizes, before and after the threshold was applied, are shown in Figure 10d. After the threshold of 100 was applied, as shown in Figure 10c, the estimated *γ*_1_ value was between 0.94 and 1.06, and the estimated *γ*_2_ and *γ*_3_ values were both between 1.14 and 1.25. We estimated the values of these exponents (averaged for the entire period) as $\gamma_{1}\approx 1.0$, $\gamma_{2}\approx 1.2$, and $\gamma_{3}\approx 1.2$. However, the slow and systematic fluctuations of the estimated *α* (black line) and *β* (red line) values, i.e., the trivariate scaling exponents defined in Equation (7), were noticeable.

Despite our use of linear regression, estimation of the uncertainty requires a nonparametric method, because the “error terms” are not normally distributed even with their homoscedasticity. Thus, we used the bootstrap method [39] to determine the confidence intervals (CIs). Resampling was performed 10,000 times, and the resampling size was identical to the sample size. The 95% CIs were determined as the 2.5- and 97.5-percentiles of the bootstrap distribution.

Considering the uncertainty of the estimations, our finding of *β* > *α* was true for all the years, as shown in Figure 12a, where the estimated scaling exponents for different years are plotted with the uncertainties based on the bootstrap method. For example, the results for 2014 were *α* = 0.49 (95% CI (0.455, 0.531)) and *β* = 0.72 (95% CI (0.697, 0.744)). Thus, we conclude that the number of employees (*ℓ*) has a higher relevance to sales *s* than the number of trading partners (*k*).

Although the changes were not large and inequality *α* < *β* was invariably satisfied, exponents *α* and *β* became smaller and larger, respectively, in the 2000–2005 period compared with the 2013–2015 period. The difference was “significant” in the sense that the 95% CIs did not overlap. Additionally, *α* and *β* were negatively correlated, which we expected from Equation (10) and the relatively robust *γ*_1_ and *γ*_2_ (Figure 10c).

**Figure 11:**
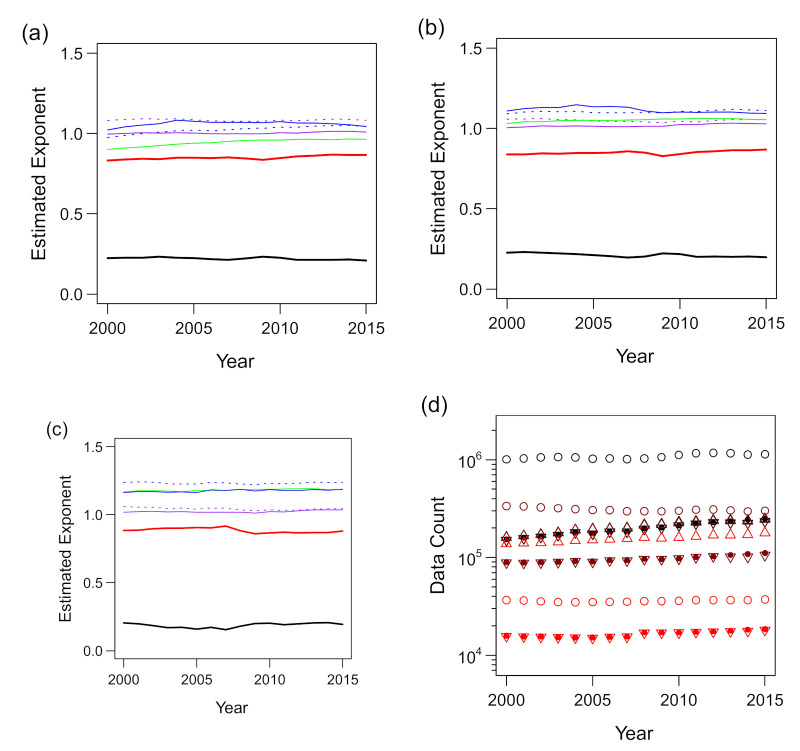
Estimated exponents of the trivariate scaling relations of *a* against *ℓ* and *s* for the period 2000–2015. Estimated exponents $\gamma_{3}$ in $s\propto \ell^{\gamma_{3}}$ (green), $\gamma_{4}$ in $s\propto \ell^{\gamma_{4}}$ (blue), $\gamma_{5}$ in $a\propto s^{\gamma_{5}}$ (purple), and $\alpha^{\prime }$ (black) and $\beta^{\prime }$ (red) in $a\propto \ell^{\alpha^{\prime }}s^{\beta^{\prime }}$ are plotted. Additionally, the expected values of $\;\gamma_{4}$ and $\;\gamma_{5}$ derived mathematically from $\alpha^{\prime }$, $\beta^{\prime }$, and $\gamma_{3}$ in the case of perfect scaling (Equations (10) and (11)) are juxtaposed (blue and purple dashed lines, respectively). (**a**) All the available data are used to estimate the exponents. (**b**) Only the data for which the “explanatory” variable is >10 are used. (**c**) Only the data for which the “explanatory” variable is >100 are used. (**d**) Plot of the size of samples from which the exponents are estimated against the year. The black, brown, and red points indicate the thresholds of 0 (no exclusion), 10, and 100, corresponding to panels (**a**–**c**), respectively. The hollow (○) and filled (●) circles and upward (△) and downward (▽) triangles represent the scaling of *s* vs. *ℓ*, *a* vs. *ℓ*, *a* vs. *s*, and *a* vs. *ℓ* and *s*, respectively.

We then applied the aforementioned procedure to the trivariate scaling of total assets *a* with *ℓ* and *s*. The results are shown in Figure 11. In contrast to the scaling of *s* with *k* and *ℓ*, there was no obvious improvement in the estimation of $\alpha^{\prime }$ and $\beta^{\prime }$ by the use of thresholding. This should be related to better fitting of the trivariate data of (*ℓ*, *s*, *a*) to the scaling surface hypothesis (see Figure 3a) compared to the (*k*, *ℓ*, *s*) data, as seen in Figure 8a and Figure 9c. The degree of the time variation of $\alpha^{\prime }$ and $\beta^{\prime }$ was noticeably smaller in the (*ℓ*, *s*, *a*) data than in the (*k*, *ℓ*, *s*) data. The estimated $\alpha^{\prime }$ (exponent for *ℓ*) and $\beta^{\prime }$ (exponent for *s*) values were between 0.17 and 0.24 and between 0.82 and 0.92, respectively, in all years and for all threshold values that we applied. In contrast, the bivariate scaling exponents were estimated without data with the *s*- or *a*-value less than 100 to exclude the data that did not conform to the power law, as seen in Figure 5b,c. Therefore, the final values of estimated *γ*_4_ (for *ℓ*–*a* scaling) and *γ*_5_ (for *s*–*a* scaling) were between 1.16 and 1.19 and between 1.01 and 1.04, respectively, as shown in Figure 11c.

### 3.6. Scaling Exponents Related to National Economic Conditions

As indicated in the previous section, there were significant changes (i.e., changes beyond the CI) in *α* and *β* during the period 1994–2015, although the source of this fluctuation remains to be explored. Here, we only test the hypothesis that national macroeconomic conditions are related to the changes in these scaling exponents.

**Figure 12:**
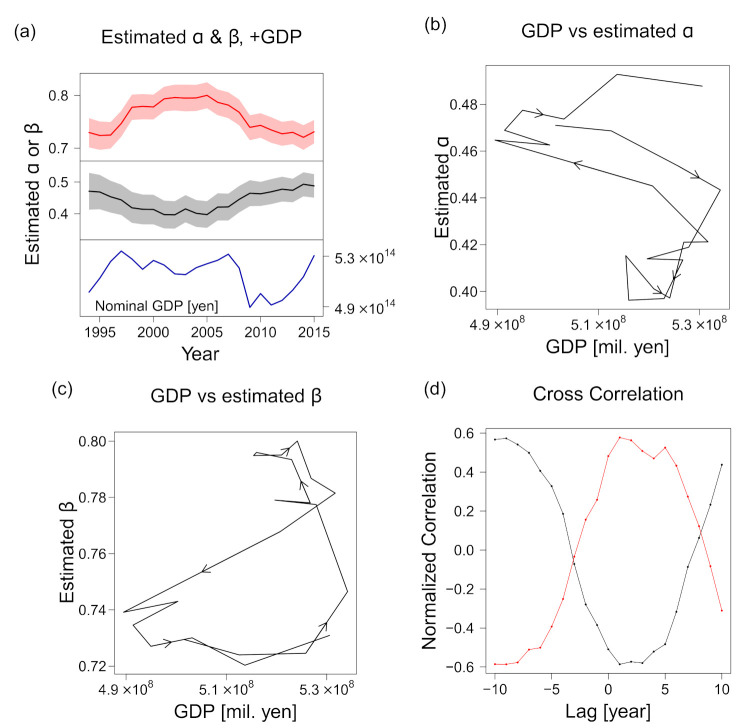
Estimated exponents *α* (for number of trading partners *k*) and *β* (for number of employees *ℓ*) with the country’s nominal GDP for different years in 1994–2015. (**a**) Estimated exponents *α* (for number of trading partners *k*) and *β* (for number of employees *ℓ*) and the country’s nominal GDP for different years in the period 1994–2015. The bandwidths indicate the 95% confidence interval of the estimation obtained via the bootstrap method. (**b**) Estimated *α* with respect to the GDP. The arrows indicate the direction of time evolution. (**c**) Estimated *β* with respect to the GDP. (**d**) Cross correlations between the nominal GDP and *α* or *β* are plotted with respect to the time lag.

Interestingly, *α* and *β* appeared counter- and pro-cyclical, respectively; i.e., they were apparently positively and negatively correlated to the nominal GDP of the country [40], respectively, as shown in Figure 12a. The GDP was selected here because its fluctuation cycle is longer than those of other economic indices, such as the Indexes of Business Conditions reported by the Cabinet Office of the Government of Japan [41]. A closer inspection revealed that *α* was enlarged when the nominal GDP decreased (Figure 12b) and that *β* decreased almost simultaneously with the GDP, whereas its increase was delayed with respect to the GDP expansion (Figure 12c).

We calculated the cross-correlation between the national GDP and the scaling exponents to evaluate the delay of the effect of the former on the latter. The normalized cross-correlation, ${\mathrm{CC}}_{\tau }\left[x,y\right],$ was defined by the Pearson’s correlation coefficient applied to lagged time-series data $x\left(t\right)$ and $y\left(t+\tau \right)$ defined for discrete time $t_{0}\leq t\left(\in \mathbb{Z}\right)\leq t_{\mathrm{end}}$:

(15) CCτx,y ≡1N−1∑Tτx˜ty˜t+τ1N−1∑Tτx˜t21N−1∑Tτy˜t+τ2 ,

where $T_{\tau }\equiv \;\left\{t\in \mathbb{Z}|t_{0}+\tau \leq t\leq t_{\mathrm{end}}+\tau \right\}$, *N* represents the length of the time series (the number of elements in truncated set $T_{\tau }$), and

(16) $$\tilde{x}\left(t\right)\;\equiv x\left(t\right)-\frac{1}{N}\sum_{T_{\tau }}x\left(t\right)\;\mathrm{and}\;\tilde{y}\left(t\right)\;\equiv y\left(t\right)-\frac{1}{N}\sum_{T_{\tau }}y\left(t\right).$$

As shown in Figure 12d, we plotted the cross correlations, ${\mathrm{CC}}_{\tau }\left[\mathrm{GDP},\hat{\alpha }\right]$ and ${\mathrm{CC}}_{\tau }\left[\mathrm{GDP},\hat{\beta }\right],$ with respect to *τ* (the time lag in years) to quantify the delay of the changes in the estimated *α* and *β* values with respect to the GDP. Both the cross correlations peaked at *τ* = 1 (negatively and positively, respectively), with Pearson correlation coefficients as large as –0.59 and 0.58. The second-highest peak was present in both cross-correlation series, indicating that the extent of the delay in the exponent changes differed between the increasing GDP and the decreasing GDP. The reversal of the sign at a *τ* value far from 0 was an artefact of the time window, which covered only slightly more than one economic cycle. The foregoing results suggest that the exponents can be predicted using the GDP of the preceding year. However, we could not eliminate the possibility that the correlations were mere coincidences, as the dataset covered only slightly more than one business cycle. Considering that the cycle was approximately 20 years long, an additional decade of data or a dataset from another country may be needed to verify this trend.

We suspect that the relative importance of inter-firm trades in predicting sales is mechanistically affected by the GDP. It is reasonable that in a recovering economy, selling goods produced with labor is relatively easy, and increasing the number of employees is advantageous for expanding production, which leads to an increase in *β*. In contrast, in an economic depression, the trading partners to which the firm can sell products may become more crucial for maintaining its sales; in such a situation, weight may be added to *α*. However, this statement is not supported by the present study and is yet to be verified.

## 4. Discussion

We analyzed the multivariate scaling relations between firm size measures. First, the marginal distributions of *k*, *ℓ*, *s*, *a*, and *p* were examined, and the former four were found to have a power–law tail while the right tail of the latter one did not exhibit a clear power law. Second, we examined the bivariate relations between the size measures to uncover that relations for pairs of *k*, *ℓ*, *s*, and *a* could all be approximated by an allometric scaling except the *k*–*a* relation, while the capital stock, *p,* was never in an allometric scaling with other size measures. Based on the empirically verified model of allometric scaling, seemingly inconsistent power–law exponents for the right tails of conditional and marginal distributions were theoretically explained. Furthermore, theoretical considerations clarified that the trivariate allometric scaling is related to the transitivity of bivariate allometric scalings. This was exemplified by the comparison of trivariate data of (*k*, *ℓ*, *s*) and (*ℓ*, *s, a*), which conformed to trivariate allometric scaling, with the data of (*k*, *ℓ*, *a*) which did not fit the allometric scaling model. Building on the theoretical development, a method was proposed and applied to the data to estimate the scaling exponents for each year. We observed only small variations when data from different years were compared. The fluctuations of the scaling exponents *α* and *β* for the data of (*k*, *ℓ*, *s*) during a 22-year period apparently followed the country’s GDP.

We argue that our results might be highly valuable in making the existing research results more robust and clearer. Some reported results regarding corporate finance were not robust when different size measures were used as a control variable indicating corporate size [19], suggesting that a single variable indicating the firm size is not sufficient to control the “size effects.” An allometric scaling between size measures, e.g., *x*∝*y^γ^*, means that the fluctuation or deviation from the average relation (*x/y^γ^*) is independent of *y*. Therefore, such an adjusted ratio between size measures in allometric scalings could be added to analyses as a possible factor improving the robustness of the existing methods against the use of alternative corporate size measures, bridging the studies using different size measures of firms as a control variable.

Scaling relations for data of more than three measures of size were not examined in our study. Although it is possible to conceive of such a theoretical development, empirical verification of the statistical model for a high-dimensional space is expected to face a difficulty known as the curse of dimensionality [42]. Furthermore, we do not expect theories regarding four-dimensional allometric scaling to be useful in understanding empirical data, since the allometric scaling was found to be broken in the three-dimensional data of (*k*, *ℓ*, *a*).

The current analyses are based on aggregated data from all industries. It is highly possible that firms in different industries or markets have different scaling relations. Nevertheless, our results would still serve as a basis for investigating the characteristics of different industries and markets, since the results can be regarded as representing average behaviors of firms across different sectors, calculated from nearly the most comprehensive dataset. Therefore, it would be a promising research direction to study the uniqueness of different groups of firms relative to the average behaviors.

It is our current expectation that similar results could be obtained if it were possible to conduct the same analysis on different datasets of business firms operating in areas other than Japan. Tests of the reproducibility of our results would be highly valuable for understanding the diversity and universality of business firms. While analysis regarding the sales, total assets, and employees of firms can be performed with minimal effort owing to the abundance of information, it would be more difficult to include the number of trading partners because data related to trading between firms are often missing in existing datasets. Text mining of published corporate reports may be a promising approach for compiling datasets of inter-firm trading [43]. We also encourage the application of our method to other systems that exhibit scale-invariant relative fluctuations, such as metropolises and cities [44,45].

We confirmed that the fluctuation of *s*—conditional to *k* and *l* and relative to the median—was invariant, directly verifying the hypothesis proposed in the previous work [23]. This scale-invariance of the relative fluctuation is highly suggestive of the similarity between firms of different sizes and the renormalizability of internal organizations or inter-firm relationships with regard to complex networks [46,47].

Verifying previously hypothesized stylized facts and checking the mathematical consistency of independently known power–law exponents help to validate or refute possible theories regarding business firms. Numerous models [35,48,49,50,51,52,53,54,55,56,57,58,59,60,61] have been suggested to explain only a few stylized facts relating to firms, and it appears that new criteria are needed to select the models empirically. Thus, our results for multivariate scaling should be incorporated into subsequent theoretical considerations. Theories for the scaling relations in other complex systems, such as animal bodies and cities, may benefit from this study because fractal-like hierarchical organization is a pervasive design in such systems (including business firms) [15,32,33,62]. However, the data are far more abundant for firms than for other systems. The multidimensional notion of size may introduce prospects for devising general theories and modeling principles for such complex systems.

## Data Availability

Restrictions apply to the availability of these data. Data was obtained from Teikoku Databank, Ltd. (Japan) and are available from the authors with the permission of Teikoku Databank, Ltd. (Japan).

## Supplementary Materials

The following are available online at https://www.mdpi.com/1099-4300/23/2/168/s1, Table S1: Descriptive statistics of the final data of firm size measures for different years.

## Appendix A. Rationales for Estimation of Scaling Exponents

Here, we consider the rationales for the estimations of the scaling exponents using the standard regression analysis.

We begin with the bivariate scaling relations, which were formulated with Equations (1)–(3) or (4)–(6) and confirmed to be present in our data:

$$P\left(x|y\right)\;={\tilde{P}}_{x|y}\left(x/y^{\gamma }\right)/y^{\gamma },$$

where $P\left(x|y\right)$ represents the probability density of *x* conditional on *y*, ${\tilde{P}}_{x|y}$ represents a probability density function, and γ is a positive constant. When variable $\tilde{x}$ is defined as $\tilde{x}\equiv x/y^{\gamma }$, we have

$$P\left(\tilde{x}|y\right)={\tilde{P}}_{x|y}\left(\tilde{x}\right),$$

given that the probability density should satisfy normalization $\int P\left(\tilde{x}|y\right)d\tilde{x}=1$. Variable $\tilde{x}$ does not depend on the *y*-value; thus, $\tilde{x}$ is independent of *y*.

This indicates that exponent γ can be estimated by finding the value that maximizes the degree of independence of $\tilde{x}$ and *y*. One of the simplest indices for measuring the dependence between stochastic variables is the Pearson’s product–moment correlation coefficient. We can obtain $\hat{\gamma }$, i.e., the estimated value of γ, as follows:

(A1) $$\hat{\gamma }=\underset{\gamma }{\arg\;\min}{\left(\mathrm{Cor}\left[\log\left(\frac{x}{y^{\gamma }}\right),\log y\right]\right)}^{2},$$

where $\mathrm{Cor}\left[\cdot ,\cdot \right]$ is the correlation coefficient of the two terms. We apply the log-transformation to the raw data because the distribution of *x* conditional on *y* is heavy-tailed, as shown in Figure 4d–f. In such heavy-tailed distributions, a few extreme values can substantially affect the correlation coefficient compared with the case of the normal or exponential distribution.

The minimization of the absolute value of correlation coefficients amounts to the linear least-squares regression of logx against logy. Indeed, when the correlation coefficient is zero, the covariance is also zero, and if we apply transformations $x\leftarrow x/\bar{x}$ and $y\leftarrow y/\bar{y}$, where $\bar{x}$ and $\bar{y}$ represent the geometric means of x and y, respectively,

$$\left(N-1\right)\times \mathrm{Cov}\left[\log\left(\frac{x}{y^{\gamma }}\right),\log y\right]\equiv \sum_{i}\log y_{i}\times \left(\log x_{i}-\gamma \log y_{i}\right)=0$$

or

(A2) $$\sum_{i}\log x_{i}\times \log y_{i}=\gamma \sum_{i}{\left(\log y_{i}\right)}^{2}.$$

Here, $\mathrm{Cov}\left[\cdot ,\cdot \right]$ represents the covariance, N represents the number of samples, and *x_i_* and *y_i_* represent the *i*^th^ samples of *x* and *y*, respectively. When the residual sum of squares is minimized,

$$\frac{\partial }{\partial \gamma }\sum_{i}{\left(\log x_{i}-\gamma \log y_{i}\right)}^{2}=0$$

should hold. This is indeed satisfied by Equation (A2).

Similar considerations are valid for the estimation of exponents in multivariate scaling. Assuming Equation (8) and defining $\tilde{s}\equiv s/k^{\alpha }l^{\beta }$, the probability function of $\tilde{s}$ is constant regardless of *k* and *ℓ*: $P\left(\tilde{s}|k,l\right)={\tilde{P}}_{s|k,l}\left(\tilde{s}\right)$. Therefore, *α* and *β* can be estimated as follows:

(A3) $$\left(\hat{\alpha },\hat{\beta }\right)=\underset{\left(\alpha ,\beta \right)}{\arg\;\min}\left[{\left(\mathrm{Cor}\left[\log\left(\frac{s}{k^{\alpha }l^{\beta }}\right),\log k\right]\right)}^{2}+{\left(\mathrm{Cor}\left[\log\left(\frac{s}{k^{\alpha }l^{\beta }}\right),\log l\right]\right)}^{2}\right].$$

When the correlation coefficients are zero so that the right-hand side of Equation (A3) is minimal and when we apply transformations $s\leftarrow s/\bar{s}$, $k\leftarrow k/\bar{k}$, and $l\leftarrow l/\bar{l}$, where $\bar{x}$ represents the geometric mean of the variable x,

$$\sum_{i}\left(\log s_{i}-\alpha \log k_{i}-\beta \log l_{i}\right)\times \log k_{i}=\sum_{i}\left(\log s_{i}-\alpha \log k_{i}-\beta \log l_{i}\right)\times \log l_{i}=0,$$

where *k_i_*, *ℓ_i_*, and *s_i_* represent the *i*th samples of *k*, *ℓ*, and *s*, respectively. Under this condition,

(A4) $$\frac{\partial }{\partial \alpha }\sum_{i}{\left(\log s_{i}-\alpha \log k_{i}-\beta \log l_{i}\right)}^{2}=0;\;\frac{\partial }{\partial \beta }\sum_{i}{\left(\log s_{i}-\alpha \log k_{i}-\beta \log l_{i}\right)}^{2}=0.$$

Therefore, the estimation in Equation (A3) can be equated to the linear least-squares regression of logs against logk and logl without the interaction term.

Although it is more typical to remove the correlations between “explanatory” variables in the regression analysis, the foregoing method provides a result equivalent to such an orthogonalized version of regression. Let us consider the regression of logs against logk and $\log\left[l/k^{\gamma_{1}}\right]$, where $\gamma_{1}$ is a constant determined empirically ($\gamma_{1}\approx 1.0$, as shown in Figure 10c). The variables are again normalized with transformation $x\leftarrow x/\bar{x}$, where $\bar{x}$ represents the geometric mean of the variable x. Here, exponents $\alpha^{\prime }$ and $\beta^{\prime }$ are intended to fulfill the multivariate scaling, $s\propto k^{\alpha^{\prime }}{\left(\frac{l}{k^{\gamma_{1}}}\right)}^{\beta^{\prime }},$ and are thus estimated as follows:

$$\left({\hat{\alpha }}^{\prime },{\hat{\beta }}^{\prime }\right)=\underset{\alpha^{\prime },\beta^{\prime }}{\arg\;\min}\left[\mathrm{Cor}{\left[\log\left(\frac{s}{k^{\alpha^{\prime }}{\left(l/k^{\gamma_{1}}\right)}^{\beta^{\prime }}}\right),\log k\right]}^{2}+\mathrm{Cor}{\left[\log\left(\frac{s}{k^{\alpha^{\prime }}{\left(l/k^{\gamma_{1}}\right)}^{\beta^{\prime }}}\right),\log\left(l/k^{\gamma_{1}}\right)\right]}^{2}\right].$$

When the correlations are zero,

(A5) $$\sum_{i}\left(\log s_{i}-\alpha^{\prime }\log k_{i}-\beta^{\prime }\left(\log l_{i}-\gamma_{1}\log k_{i}\right)\right)\cdot \log k_{i}=0;$$

(A6) $$\sum_{i}\left(\log s_{i}-\alpha^{\prime }\log k_{i}-\beta^{\prime }\left(\log l_{i}-\gamma_{1}\log k_{i}\right)\right)\cdot \left(\log l_{i}-\gamma_{1}\log k_{i}\right)=0.$$

By adding Equation (A6) to Equation (A5) multiplied by $\;\gamma_{1}$, we obtain

(A7) $$\sum_{i}\left(\log s_{i}-\alpha^{\prime }\log k_{i}-\beta^{\prime }\left(\log l_{i}-\gamma_{1}\log k_{i}\right)\right)\cdot \log l_{i}=0.$$

Equations (A5) and (A7) are satisfied with $\alpha^{\prime }=\alpha +\beta \gamma_{1}$ and $\beta^{\prime }=\beta$ when Equation (A4) holds. Therefore, the estimated values of the exponents in a formal regression analysis can be derived from the regression with the “explanatory” variables (not orthogonalized). Note that $\alpha^{\prime }$ is the mathematically expected value of $\gamma_{2}$, as indicated by Equation (A16) in Appendix B.

## Appendix B. Derivation of Asymptotic Power–Law or Scaling Exponents

Here, we derive the relations between asymptotic exponents for power–law distributions and scalings assuming the bi- and trivariate scaling relations. This allows us to check the consistency of results of regression analyses. First, given Equations (1)–(3), we derive the asymptotic power–law exponent of the marginal distribution, $P\left(x\right),$ of the “explained” variable, *x*. Second, given Equations (1) and (8), we calculate the asymptotic power–law exponent of the marginal distribution of *s*. Third, we show that the bivariate scaling exponents, *γ*_2_ and *γ*_3_ (i.e., those of sales *s* against number of trading partners *k* and against number of employees *ℓ*, respectively), can be determined from *α*, *β*, and *γ*_1_.

Because Equations (1)–(3) have the general form:

(A8) $$P\left(x|y\right)\;={\tilde{P}}_{x|y}\left(x/y^{\gamma }\right)/y^{\gamma }$$

we can derive the power–law exponent at the tail of the density function of the marginal distribution, $P_{x}\left(x\right)\;\equiv \;P\left(x\right),$ for variable *x* given the following: positive scaling exponent *γ*, ${\tilde{P}}_{x|y}$ (the universal distribution function of *x* conditional on *y*), and marginal distribution $P_{y}\left(y\right)\;\equiv P\left(y\right)$. Assuming that ${\tilde{P}}_{x|y}$ and $P_{y}$ have power–law right tails of exponents $\delta_{x|y}$ and $\delta_{y}$, respectively, we approximate the functions as

(A9) $${\tilde{P}}_{x|y}\left(\tilde{x}\right)=\left\{\begin{array}{ll} c_{x|y}{\tilde{x}}^{-\delta_{x|y}} & \;\;\;\left(\tilde{x}\geq 1\right)\\ d_{x|y}\left(\tilde{x}\right) & \left(0\leq \tilde{x}<1\right) \end{array}\right.$$

and

(A10) $$P_{y}\left(y\right)=\left\{\begin{array}{ll} c_{y}y^{-\delta_{y}} & \;\;\;\left(y\geq 1\right)\\ d_{y}\left(y\right) & \left(0\leq y<1\right) \end{array}\right.$$

where $c_{x|y}$ and $c_{y}$ are positive constants, $\delta_{x|y}$ and $\delta_{y}$ represent the power–law exponents larger than 1, and $d_{x|y}$ and $d_{y}$ represent the distribution functions for the smaller side. This approximation is based on the actual distributions of the size variables (Figure 2a–d, Figure 4d–f and Figure 5d–f). We set the threshold beyond which the distribution follows the power law to unity because the same results are easily derived for the asymptotic value of exponents with linear transformations as long as the threshold is positive. By applying Bayes’ theorem and assuming that x>1 (because we are interested in the “tails” or asymptotic behaviors as x→∞), we derive $P_{x}\left(x\right)$, i.e., the marginal distribution of *x*, from $P\left(x,y\right)$, i.e., the joint distribution of *x* and *y*:

$$P_{x}\left(x\right)\;=\int_{0}^{\infty }P\left(x,y\right)dy=\int_{0}^{\infty }P\left(x|y\right)P\left(y\right)dy=\left(I_{1}-C\right)x^{-\delta_{x|y}}+\left(I_{2}+C\right)x^{-1-\left(\delta_{y}-1\right)/\gamma },$$

where $I_{1}$, $I_{2}$, and C are constants respectively defined as

$$I_{1}=c_{x|y}\int_{0}^{1}y^{\gamma \left(\delta_{x|y}-1\right)}d_{y}\left(y\right)dy,$$

$$I_{2}=c_{y}\int_{1}^{\infty }{\tilde{y}}^{-\gamma -\delta_{y}}d_{x|y}\left(\frac{1}{{\tilde{y}}^{\gamma }}\right)d\tilde{y},$$

$$C=c_{x|y}c_{y}/\left(\gamma \left(\delta_{x|y}-1\right)-\delta_{y}+1\right)$$

and substitution $\tilde{y}=y/x^{1/\gamma }$ is applied in the last integral term. Therefore, the asymptotic behavior of $P_{x}\left(x\right)$ at positive infinity is determined by whether $\delta_{x|y}$ or $1+\left(\delta_{y}-1\right)/\gamma$ is smaller: as x→∞, the behavior of $P_{x}$ is approximated as

(A11) $$P_{x}\left(x\right)\;\propto x^{-\delta_{x}}$$

where

(A12) $$\delta_{x}=\min\left[\delta_{x|y},\;\;\;1+\frac{\delta_{y}-1}{\gamma }\right]$$

The change in $\delta_{x|y}$ does not affect the power–law exponent as long as $\delta_{y}$ is sufficiently large; this is consistent with the intuitive explanation (Figure 7) presented in the main text.

Although rather complex, a similar strategy works for the case of multivariate scaling. Let us continue with the foregoing notations, except that *x*, *y*, and *γ* are replaced with *ℓ*, *k*, and *γ*_1_, respectively, and Equation (8) is assumed as well as the following:

(A13) $${\tilde{P}}_{s|k,l}\left(\tilde{s}\right)=\{\begin{array}{ll} c_{s|k,l}{\tilde{s}}^{-\delta_{s|k,l}} & \;\;\;\;\;\;\left(\tilde{s}\geq 1\right)\\ d_{s|k,l}\left(\tilde{s}\right) & \;\;\;\left(0\leq \tilde{s}<1\right) \end{array},$$

where $c_{s|l,k}$ is a positive constant, $\delta_{s|k,l}$ represents a power–law exponent larger than 1, and $d_{s|k,l}$ represents the distribution function for the smaller side. Again, the threshold of the functional change of ${\tilde{P}}_{s|k,l}$ is set to unity without loss of generality. The marginal distribution of *s*, i.e., $P_{s}\left(s\right)$, for s>1 is

$$\begin{array}{ll} P_{s}\left(s\right) & =\int_{0}^{\infty }\int_{0}^{\infty }P\left(k,l,s\right)dldk=\int_{0}^{\infty }\int_{0}^{\infty }P\left(s|k,l\right)P\left(k,l\right)dldk\\ & =\int_{0}^{\infty }\int_{0}^{\infty }P\left(s|k,l\right)P\left(l|k\right)P\left(k\right)dldk. \end{array}$$

This can be evaluated as the sum of the following 10 integral terms:

$$\int_{0}^{1}\int_{0}^{k^{\gamma_{1}}}P\left(s|k,l\right)P\left(l|k\right)P\left(k\right)dldk=\left[c_{s|k,l}\int_{0}^{1}k^{\left(\alpha +\beta \gamma_{1}\right)\left(\delta_{s|k,l}-1\right)}d_{k}\left(k\right)\int_{0}^{1}{\tilde{l}}^{\beta \left(\delta_{s|k,l}-1\right)}d_{l|k}\left(\tilde{l}\right)d\tilde{l}dk\right]s^{-\delta_{s|k,l}},$$

where substitution $\tilde{l}=l/k^{\gamma_{1}}$ is applied;

$$\int_{0}^{1}\int_{k^{\gamma_{1}}}^{{\left({\frac{s}{k}}^{\alpha }\right)}^{\frac{1}{\beta }}}P\left(s|k,l\right)P\left(l|k\right)P\left(k\right)dldk=\frac{c_{l|k}c_{s|k,l}}{E_{1}}\left(\left[\int_{0}^{1}k^{\left(\alpha /\beta +\gamma_{1}\right)\left(\delta_{l|k}-1\right)}d_{k}\left(k\right)dk\right]s^{-1-\left(\delta_{l|k}-1\right)/\beta }-\left[\int_{0}^{1}k^{\left(\alpha +\beta \gamma_{1}\right)\left(\delta_{s|k,l}-1\right)}d_{k}\left(k\right)dk\right]s^{-\delta_{s|k,l}}\right)$$

where $E_{1}=\beta \left(\delta_{s|k,l}-1\right)-\delta_{l|k}+1$;

$$\int_{0}^{1}\int_{{\left({\frac{s}{k}}^{\alpha }\right)}^{\frac{1}{\beta }}}^{\infty }P\left(s|k,l\right)P\left(l|k\right)P\left(k\right)dldk=\left[c_{l|k}\int_{0}^{1}k^{\left(\alpha /\beta +\gamma_{1}\right)\left(\delta_{l|k}-1\right)}d_{k}\left(k\right)dk\int_{1}^{\infty }d_{s|k,l}\left(\frac{1}{{\tilde{l}}^{\beta }}\right){\tilde{l}}^{-\delta_{l|k}-\beta }d\tilde{l}\right]s^{-1-\left(\delta_{l|k}-1\right)/\beta },$$

where substitution $\tilde{l}=\;\left(k^{\alpha /\beta }/s^{1/\beta }\right)\;\times l$ is applied;

$$\int_{1}^{s^{1/\left(\alpha +\beta \gamma_{1}\right)}}\int_{0}^{k^{\gamma_{1}}}P\left(s|k,l\right)P\left(l|k\right)P\left(k\right)dldk=\frac{c_{k}c_{s|k,l}}{E_{2}}\left[\int_{0}^{1}{\tilde{l}}^{\beta \left(\delta_{s|k,l}-1\right)}d_{l|k}\left(\tilde{l}\right)d\tilde{l}\right]\left(s^{-1-\left(\delta_{k}-1\right)/\left(\alpha +\beta \gamma_{1}\right)}-s^{-\delta_{s|k,l}}\right),$$

where substitution $\tilde{l}=l/k^{\gamma_{1}}$ is applied and $E_{2}=\left(\alpha +\beta \gamma_{1}\right)\left(\delta_{s|k,l}-1\right)-\delta_{k}+1$;

$$\int_{1}^{s^{\frac{1}{\left(\alpha +\beta \gamma_{1}\right)}}}\int_{k^{\gamma_{1}}}^{{\left({\frac{s}{k}}^{\alpha }\right)}^{\frac{1}{\beta }}}P\left(s|k,l\right)P\left(l|k\right)P\left(k\right)dldk=\frac{c_{k}c_{l|k}c_{s|k,l}}{E_{1}}\left(\left(\frac{1}{E_{3}}-\frac{1}{E_{2}}\right)s^{-1-\left(\delta_{k}-1\right)/\left(\alpha +\beta \gamma_{1}\right)}-\frac{1}{E_{3}}s^{-1-\left(\delta_{l|k}-1\right)/\beta }+\frac{1}{E_{2}}s^{-\delta_{s|k,l}}\right)$$

where $E_{3}=\left(\alpha /\beta +\gamma_{1}\right)\left(\delta_{l|k}-1\right)-\delta_{k}+1$;

$$\int_{1}^{s^{1/\left(\alpha +\beta \gamma_{1}\right)}}\int_{{\left(s/k^{\alpha }\right)}^{1/\beta }}^{\infty }P\left(s|k,l\right)P\left(l|k\right)P\left(k\right)dldk=\left[\frac{c_{k}c_{l|k}}{E_{3}}\int_{1}^{\infty }d_{s|k,l}\left(\frac{1}{{\tilde{l}}^{\beta }}\right){\tilde{l}}^{-\delta_{l|k}-\beta }d\tilde{l}\right]\left(s^{-1-\left(\delta_{k}-1\right)/\left(\alpha +\beta \gamma_{1}\right)}-s^{-1-\left(\delta_{l|k}-1\right)/\beta }\right),$$

where substitution $\tilde{l}=\left(k^{\alpha /\beta }/s^{1/\beta }\right)\times l$ is applied;

$$\int_{0}^{s^{\gamma_{1}/\left(\alpha +\beta \gamma_{1}\right)}}\int_{s^{1/\left(\alpha +\beta \gamma_{1}\right)}}^{{\left(s/l^{\beta }\right)}^{1/\alpha }}P\left(s|k,l\right)P\left(l|k\right)P\left(k\right)dkdl=\left(c_{k}c_{s|k,l}I_{1}^{3}\right)s^{-1-\left(\delta_{k}-1\right)/\left(\alpha +\beta \gamma_{1}\right)},$$

where

$$I_{1}^{3}=\int_{0}^{1}{\tilde{l}}^{-1+\left(\delta_{s|k,l}-1\right)\left(\alpha +\beta \gamma_{1}\right)/\gamma_{1}-\left(\delta_{k}-1\right)/\gamma_{1}}\int_{{\tilde{l}}^{-1/\gamma_{1}}}^{{\tilde{l}}^{-\left(\alpha +\beta \gamma_{1}\right)/\alpha \gamma_{1}}}{\tilde{k}}^{\alpha \left(\delta_{s|k,l}-1\right)-\gamma_{1}-\delta_{k}}d_{l|k}\left(\frac{1}{{\tilde{k}}^{\gamma_{1}}}\right)d\tilde{k}d\tilde{l}$$

and substitutions $\tilde{k}=l^{-1/\gamma_{1}}\times k$ and $\tilde{l}=s^{-\gamma_{1}/\left(\alpha +\beta \gamma_{1}\right)}\times l$ are applied;

$$\int_{0}^{s^{\gamma_{1}/\left(\alpha +\beta \gamma_{1}\right)}}\int_{{\left(s/l^{\beta }\right)}^{1/\alpha }}^{\infty }P\left(s|k,l\right)P\left(l|k\right)P\left(k\right)dkdl=\left(c_{k}I_{2}^{3}\right)s^{-1-\left(\delta_{k}-1\right)/\left(\alpha +\beta \gamma_{1}\right)},$$

where

$$I_{2}^{3}=\int_{0}^{1}{\tilde{l}}^{\left(\gamma_{1}+\delta_{k}-1\right)\beta /\alpha }\int_{1}^{\infty }\frac{1}{{\tilde{k}}^{\alpha }}d_{s|k,l}\left(\frac{1}{{\tilde{k}}^{\alpha }}\right)\frac{1}{{\tilde{k}}^{\gamma_{1}}}d_{s|k,l}\left(\frac{{\tilde{l}}^{\left(\alpha +\beta \gamma_{1}\right)/\alpha }}{{\tilde{k}}^{\gamma_{1}}}\right){\tilde{k}}^{-\delta_{k}}d\tilde{k}d\tilde{l}$$

and substitutions $\tilde{k}={\left(l^{\beta }/s\right)}^{1/\alpha }\times k$ and $\tilde{l}=s^{-\gamma_{1}/\left(\alpha +\beta \gamma_{1}\right)}\times l$ are applied;

$$\int_{s^{\gamma_{1}/\left(\alpha +\beta \gamma_{1}\right)}}^{\infty }\int_{l^{1/\gamma_{1}}}^{\infty }P\left(s|k,l\right)P\left(l|k\right)P\left(k\right)dkdl=\left(c_{k}I_{3}^{3}\right)s^{-1-\left(\delta_{k}-1\right)/\left(\alpha +\beta \gamma_{1}\right)},$$

where

$$I_{3}^{3}=\int_{1}^{\infty }{\tilde{l}}^{-1-\left(\alpha +\beta \gamma_{1}+\delta_{k}-1\right)/\gamma_{1}}\int_{1}^{\infty }\frac{1}{{\tilde{k}}^{\alpha }}d_{s|k,l}\left(\frac{1}{{\tilde{k}}^{\alpha }{\tilde{l}}^{\left(\alpha +\beta \gamma_{1}\right)/\gamma_{1}}}\right)\frac{1}{{\tilde{k}}^{\gamma_{1}}}d_{s|k,l}\left(\frac{1}{{\tilde{k}}^{\gamma_{1}}}\right){\tilde{k}}^{-\delta_{k}}d\tilde{k}d\tilde{l}$$

and substitutions $\tilde{k}=l^{-1/\gamma_{1}}\times k$ and $\tilde{l}=s^{-\gamma_{1}/\left(\alpha +\beta \gamma_{1}\right)}\times l$ are applied;

$$\int_{s^{\gamma_{1}/\left(\alpha +\beta \gamma_{1}\right)}}^{\infty }\int_{s^{1/\left(\alpha +\beta \gamma_{1}\right)}}^{l^{1/\gamma_{1}}}P\left(s|k,l\right)P\left(l|k\right)P\left(k\right)dkdl=\left(c_{k}c_{l|k}I_{4}^{3}\right)s^{-1-\left(\delta_{k}-1\right)/\left(\alpha +\beta \gamma_{1}\right)},$$

where

$$I_{4}^{3}=\int_{1}^{\infty }{\tilde{l}}^{-1-\left(\alpha +\beta \gamma_{1}+\delta_{k}-1\right)/\gamma_{1}}\int_{{\tilde{l}}^{-1/\gamma_{1}}}^{1}\frac{1}{{\tilde{k}}^{\alpha }}d_{s|k,l}\left(\frac{1}{{\tilde{k}}^{\alpha }{\tilde{l}}^{\left(\alpha +\beta \gamma_{1}\right)/\gamma_{1}}}\right){\tilde{k}}^{\gamma_{1}\left(\delta_{l|k}-1\right)-\delta_{k}}d\tilde{k}d\tilde{l}$$

and substitutions $\tilde{k}=l^{-1/\gamma_{1}}\times k$ and $\tilde{l}=s^{-\gamma_{1}/\left(\alpha +\beta \gamma_{1}\right)}\times l$ are applied. The sum of these 10 terms has the form

$$P_{s}\left(s\right)\;=A_{1}\times s^{-\delta_{s|k,l}}+A_{2}\times s^{-1-\frac{\delta_{l|k}-1}{\beta }}+A_{3}\times s^{-1-\frac{\delta_{k}-1}{\alpha +\beta \gamma_{1}}},$$

where $A_{1}$, $A_{2}$, and $A_{3}$ are constants independent of *s*. Therefore, the asymptotic behavior of $P_{s}$ as s→∞ is expressed as follows:

(A14) $$P_{s}\left(s\right)\propto s^{-\delta_{s}},$$

where $\delta_{s}$ is the smallest of the three exponents:

(A15) $$\delta_{s}=\min\left[\delta_{s|k,l},\;\;\;1+\frac{\delta_{l|k}-1}{\beta },\;\;\;1+\frac{\delta_{k}-1}{\alpha +\beta \gamma_{1}}\right]$$

Finally, we evaluate the values of exponents *γ*_2_ and *γ*_3_ using *α*, *β*, and *γ*_1_, given Equations (1) and (7). Then, the joint distribution of *k*, *ℓ*, and *s* is given as follows:

$$P\left(k,l,s\right)=P\left(s|k,l\right)P\left(l|k\right)P\left(k\right)=\frac{1}{k^{\alpha }l^{\beta }}{\tilde{P}}_{s|k,l}\left(\frac{s}{k^{\alpha }l^{\beta }}\right)\frac{1}{k^{\gamma_{1}}}{\tilde{P}}_{l|k}\left(\frac{l}{k^{\gamma_{1}}}\right)P_{k}\left(k\right).$$

We can obtain the joint distribution of *k* and *s* by integrating this probability with respect to *ℓ*.

$$P\left(k,s\right)=\int_{-\infty }^{\infty }P\left(k,l,s\right)dl=\int_{0}^{\infty }\frac{1}{k^{\alpha }l^{\beta }}{\tilde{P}}_{s|k,l}\left(\frac{s}{k^{\alpha }l^{\beta }}\right)\frac{1}{k^{\gamma_{1}}}{\tilde{P}}_{l|k}\left(\frac{l}{k^{\gamma_{1}}}\right)P_{k}\left(k\right)dl.$$

Comparing $P\left(k=k_{0},\;s\right)$ and $P\left(k=k_{1},\;s\right)$ we have

$$\begin{array}{ll} \text{P}\left(k=k_{1},\;s\right) & =\int_{0}^{\infty }\frac{1}{{k_{1}}^{\alpha }l^{\beta }}{\tilde{P}}_{s|k,l}\left(\frac{s}{{k_{1}}^{\alpha }l^{\beta }}\right)\frac{1}{{k_{1}}^{\gamma_{1}}}{\tilde{P}}_{l|k}\left(\frac{l}{{k_{1}}^{\gamma_{1}}}\right)P_{k}\left(k_{1}\right)\text{d}l\\ & ={\left(\frac{k_{0}}{k_{1}}\right)}^{\alpha +\beta \gamma_{1}}\times \frac{P_{k}\left(k_{1}\right)}{P_{k}\left(k_{0}\right)}\int_{0}^{\infty }\frac{1}{{k_{0}}^{\alpha }{\tilde{l}}^{\beta }}{\tilde{P}}_{s|k,l}\left(\frac{s}{{k_{0}}^{\alpha }{\tilde{l}}^{\beta }}\times {\left(\frac{k_{0}}{k_{1}}\right)}^{\alpha +\beta \gamma_{1}}\right)\frac{1}{k_{0}{}^{\gamma_{1}}}{\tilde{P}}_{l|k}\left(\frac{\tilde{l}}{k_{0}{}^{\gamma_{1}}}\right)P_{k}\left(k_{0}\right)\text{d}\tilde{l}\\ & ={\left(\frac{k_{0}}{k_{1}}\right)}^{\alpha +\beta \gamma_{1}}\times \frac{P_{k}\left(k_{1}\right)}{P_{k}\left(k_{0}\right)}\times \text{P}\left(k=k_{0},,s={\left(\frac{k_{0}}{k_{1}}\right)}^{\alpha +\beta \gamma_{1}}s\right) \end{array}$$

Therefore,

$$P\left(s|k=k_{1}\right)=\frac{1}{k_{1}{}^{\alpha +\beta \gamma_{1}}}{\tilde{P}}_{s|k}\left(\frac{s}{k_{1}{}^{\alpha +\beta \gamma_{1}}}\right),$$

where

$${\tilde{P}}_{s|k}\left(\tilde{s}\right)=\frac{k_{0}{}^{\alpha +\beta \gamma_{1}}}{P_{k}\left(k_{0}\right)}\times P\left(k=k_{0},\;\;\;s=k_{0}{}^{\alpha +\beta \gamma_{1}}\times \tilde{s}\right).$$

Comparing the foregoing result with Equation (2), we have

(A16) $$\gamma_{2}=\alpha +\beta \gamma_{1}$$

Similarly, the joint distribution of *ℓ* and *s* can be evaluated as

$$\text{P}\left(l=l_{0},\;s\right)\;=\int_{-\infty }^{\infty }\text{P}\left(k,\;l=l_{0},\;s\right)\text{d}k=\int_{0}^{\infty }\frac{1}{k^{\alpha }l_{0}{}^{\beta }}{\tilde{P}}_{s|k,l}\left(\frac{s}{k^{\alpha }l_{0}{}^{\beta }}\right)\frac{1}{k^{\gamma_{1}}}{\tilde{P}}_{l|k}\left(\frac{l}{k^{\gamma_{1}}}\right)P_{k}\left(k\right)\text{d}k.$$

If we assume a power–law distribution of *k* similar to Equation (A10), i.e.,

$$P_{k}\left(k\right)=\left\{\begin{array}{ll} c_{k}k^{-\delta_{k}} & \;\;\;\left(y\geq \theta_{k}\right)\\ d_{k}\left(k\right) & \left(0\leq y<\theta_{k}\right) \end{array}\right.$$

where *θ_k_* is a positive constant of the threshold, then by comparing $P\left(l=l_{0},s\right)$ with $P\left(l=l_{1},s\right)$ we obtain

$$\begin{array}{ll} \text{P}\left(l=l_{1},\;s\right)= & {\left(\frac{l_{0}}{l_{1}}\right)}^{\left(\gamma_{1}-1+\alpha +\beta \gamma_{1}\right)/\gamma_{1}}[{\left(\frac{l_{0}}{l_{1}}\right)}^{\delta_{k}/\gamma_{1}}\text{P}\left(l=l_{0},\;{\left(\frac{l_{0}}{l_{1}}\right)}^{\left(\alpha +\beta \gamma_{1}\right)/\gamma_{1}}s\right)\\ & -\int_{0}^{\theta_{k}}\frac{1}{{\tilde{k}}^{\alpha }l_{0}{}^{\beta }}{\tilde{P}}_{s|k,l}\left(\frac{s}{{\tilde{k}}^{\alpha }l_{0}{}^{\beta }}\times {\left(\frac{l_{0}}{l_{1}}\right)}^{\left(\alpha +\beta \gamma_{1}\right)/\gamma_{1}}\right)\frac{1}{{\tilde{k}}^{\gamma_{1}}}{\tilde{P}}_{l|k}\left(\frac{l_{0}}{{\tilde{k}}^{\gamma_{1}}}\right)P_{k}\left(\tilde{k}\right)\text{d}\tilde{k}]\\ & +\int_{0}^{\theta_{k}{\left(l_{1}/l_{0}\right)}^{1/\gamma_{1}}}\frac{1}{k^{\alpha }l_{1}{}^{\beta }}{\tilde{P}}_{s|k,l}\left(\frac{s}{k^{\alpha }l_{1}{}^{\beta }}\right)\frac{1}{k^{\gamma_{1}}}{\tilde{P}}_{l|k}\left(\frac{l}{k^{\gamma_{1}}}\right)P_{k}\left(k\right)\text{d}k. \end{array}$$

Here, substitution $\tilde{k}={\left(l_{0}/l_{1}\right)}^{1/\gamma_{1}}\times k$ is used. Clearly, the two integral terms in this strict relation becomes negligible as s→∞. Under an additional condition that Equation (A11) holds for l with $–\delta_{l}=-1-\left(\delta_{k}-1\right)/\gamma_{1}$

$$P\left(s|l=l_{1}\right)=\frac{P\left(l=l_{1},s\right)}{P\left(l=l_{1}\right)}\sim \frac{1}{l_{1}{}^{\left(\alpha +\beta \gamma_{1}\right)/\gamma_{1}}}{\tilde{P}}_{s|l}\left(\frac{s}{l_{1}{}^{\left(\alpha +\beta \gamma_{1}\right)/\gamma_{1}}}\right)$$

as $l_{1}\to \infty$ and $s/l_{1}{}^{\left(\alpha +\beta \gamma_{1}\right)/\gamma_{1}}\to \infty$ where

$${\tilde{P}}_{s|l}\left(\tilde{s}\right)=l_{0}{}^{\left(\alpha +\beta \gamma_{1}\right)/\gamma_{1}}\times P\left(l=l_{0},s=l_{0}{}^{\left(\alpha +\beta \gamma_{1}\right)/\gamma_{1}}\times \tilde{s}\right).$$

Comparing this result with Equation (3), we have

(A17) $$\gamma_{3}=\frac{\alpha +\beta \gamma_{1}}{\gamma_{1}}.$$

for the right tail of *s* in a limited condition.
