# Supplementary material for: Robust Characterization of Multidimensional Scaling Relations between Size Measures for Business Firms
Source: Entropy (Basel). 2021 Jan 29;23(2):168. doi: 10.3390/e23020168 (PMC7910913; doi:10.3390/e23020168)
Supplement: Supplementary file 1 [file entropy-23-00168-s001.pdf]

Table S1. Descriptive statistics of the final data of firm size measures. Decimal parts are rounded in cases where the figure exceeds four digits.

Variable	Year	Sample size	Mean	Standard deviation	Minimum	25%-quantile	Median	75%-quantile	Maximum	Interquartile range	Geometric mean	Geometric stand. dev.
Number of Trading Partners	1994	770311	4.740	31.46	1	1	2	4	7881	3	2.498	2.335
	1995	803488	4.979	32.40	1	1	2	4	8013	3	2.618	2.359
	1996	831859	5.182	33.03	1	1	3	5	8009	4	2.722	2.375
	1997	857350	5.395	33.43	1	1	3	5	7945	4	2.826	2.398
	1998	882769	5.532	33.48	1	2	3	5	7696	3	2.908	2.403
	1999	923288	5.615	33.21	1	2	3	5	7393	3	2.961	2.407
	2000	950796	5.727	33.30	1	2	3	5	7082	3	3.018	2.415
	2001	973409	5.854	33.42	1	2	3	5	6864	3	3.080	2.429
	2002	997949	5.911	34.09	1	2	3	5	6424	3	3.100	2.437
	2003	1009667	5.963	33.16	1	2	3	5	6007	3	3.118	2.456
	2004	1009889	6.071	33.32	1	2	3	5	5832	3	3.163	2.472
	2005	1006855	6.208	34.19	1	2	3	6	5562	4	3.225	2.484
	2006	995651	6.341	36.30	1	2	3	6	10987	4	3.277	2.501
	2007	996413	6.468	38.22	1	2	3	6	14941	4	3.324	2.519
	2008	1009188	6.837	39.81	1	2	3	6	15587	4	3.474	2.570
	2009	1026513	6.934	39.87	1	2	3	7	15504	5	3.512	2.593
	2010	1064424	6.885	39.94	1	2	3	7	15466	5	3.467	2.607
	2011	1082665	6.881	40.10	1	2	3	7	15252	5	3.456	2.615
	2012	1086584	6.893	40.59	1	2	3	7	15161	5	3.455	2.622
	2013	1090161	6.958	41.29	1	2	3	7	14928	5	3.487	2.627
	2014	1098323	7.003	41.14	1	2	3	7	14895	5	3.500	2.637
	2015	1097372	7.052	40.93	1	2	3	7	14740	5	3.535	2.639

(Continued)

Table S1. Continued.

Variable	Year	Sample size	Mean	Standard deviation	Minimum	25%-quantile	Median	75%-quantile	Maximum	Interquartile range	Geometric mean	Geometric stand. dev.
Number of Employees	1994	936043	29.88	380.0	1	3	7	18	208028	15	8.152	3.760
	1995	949379	29.31	366.2	1	3	7	18	191436	15	8.027	3.754
	1996	970083	28.73	356.4	1	3	7	18	186706	15	7.916	3.736
	1997	979906	28.29	335.4	1	3	7	17	161488	14	7.799	3.738
	1998	994971	27.50	292.1	1	3	7	16	77877	13	7.568	3.737
	1999	1023699	26.31	281.6	1	3	6	15	77033	12	7.207	3.718
	2000	1037670	25.69	277.7	1	3	6	15	84242	12	6.983	3.716
	2001	1062192	24.74	267.7	1	3	6	15	91026	12	6.739	3.698
	2002	1085096	23.71	255.0	1	3	5	14	97474	11	6.440	3.682
	2003	1097615	23.06	247.4	1	2	5	13	100090	11	6.230	3.679
	2004	1089568	23.07	341.7	1	2	5	13	271368	11	6.138	3.694
	2005	1057508	23.62	341.9	1	2	5	13	261937	11	6.137	3.743
	2006	1062881	23.54	336.2	1	2	5	13	256572	11	6.105	3.737
	2007	1044619	24.16	365.7	1	2	5	13	254177	11	6.084	3.763
	2008	1057914	24.17	313.6	1	2	5	13	143276	11	5.997	3.768
	2009	1088585	23.69	307.9	1	2	5	12	140846	10	5.817	3.751
	2010	1144634	22.88	301.9	1	2	5	12	136906	10	5.622	3.724
	2011	1194421	22.28	291.2	1	2	5	11	139320	9	5.519	3.707
	2012	1204466	22.24	326.0	1	2	5	11	209000	9	5.465	3.712
	2013	1206579	22.28	321.5	1	2	5	11	200601	9	5.453	3.723
	2014	1219234	22.45	326.5	1	2	4	11	194688	9	5.436	3.733
	2015	1215263	22.76	327.6	1	2	5	11	193934	9	5.469	3.757

(Continued)

Table S1. Continued.

Variable	Year	Sample size	Mean	Standard deviation	Minimum	25%-quantile	Median	75%-quantile	Maximum	Interquartile range	Geometric mean	Geometric stand. dev.
Annual Sales [$\times 10^6$ yen]	1994	1008122	1287	43135	1	70	180	475	16134997	405	197.1	4.499
	1995	1027730	1261	40821	1	70	180	463	15942401	393	193.7	4.514
	1996	1043189	1301	42242	1	70	177	465	15491756	395	192.9	4.550
	1997	1060980	1301	39472	1	70	173	460	14176418	390	190.0	4.608
	1998	1092909	1224	37819	1	62	159	422	14465555	360	174.9	4.623
	1999	1125046	1121	32808	1	58	145	390	12372623	332	160.3	4.602
	2000	1149651	1103	31164	1	55	137	370	10658978	315	152.2	4.653
	2001	1181751	1079	30370	1	50	127	350	10927418	300	142.7	4.722
	2002	1205695	1011	27694	1	46	118	320	9562842	274	131.6	4.750
	2003	1215605	992.4	26577	1	44	110	306	9419359	262	125.2	4.802
	2004	1212376	1025	34296	1	42	109	301	24602332	259	123.3	4.863
	2005	1200578	1063	33110	1	41	108	304	20633322	263	123.0	4.925
	2006	1194911	1107	36071	1	40	106	305	23061200	265	122.2	5.000
	2007	1199061	1141	35130	1	40	104	303	19604000	263	120.5	5.082
	2008	1222606	1140	32924	1	39	100	300	12291218	261	114.0	5.198
	2009	1287709	1015	29615	1	32	90	259	11130100	227	98.49	5.295
	2010	1347533	899.6	24955	1	30	80	234	9656263	204	89.27	5.297
	2011	1379216	908.5	26313	1	30	80	229	10151462	199	86.32	5.363
	2012	1396227	907.8	26931	1	30	80	227	10454663	197	85.43	5.405
	2013	1387639	914.0	28006	1	29	79	221	10781950	192	84.54	5.414
	2014	1344994	977.6	30476	1	29	80	230	11042163	201	85.74	5.532
	2015	1360922	974.2	28766	1	29	80	231	11244832	202	86.10	5.558

(Continued)

Table S1. Continued.

Variable	Year	Sample size	Mean	Standard deviation	Minimum	25%-quantile	Median	75%-quantile	Maximum	Interquartile range	Geometric mean	Geometric stand. dev.
Total Assets [$\times 10^3$ yen]	2000	181686	4135493	74423716	10	81683	255329	882316	14294811000	800633	290191	6.328
	2001	186858	4104281	74911398	3	72828	236892	841968	14297626000	769140	268857	6.563
	2002	189894	3894099	72769318	2	67852	222058	799188	14174834000	731337	252077	6.604
	2003	194364	3694630	70104721	32	64209	209452	760592	13812538000	696382	239379	6.595
	2004	207723	3467672	67685487	31	56705	184483	680718	13434326000	624013	213927	6.630
	2005	213294	3429830	66719176	30	55760	182463	669589	13101186000	613829	209787	6.667
	2006	220572	3471718	68552052	3	53451	178874	657628	13031464000	604177	203486	6.771
	2007	224971	3510722	69913838	11	51631	175528	652767	12924022000	601136	199031	6.882
	2008	238993	3299829	68482441	10	45719	156947	610713	13057731000	564994	181016	7.001
	2009	237668	3162473	66969138	2	44078	151853	588000	12990060000	543922	172869	7.018
	2010	246010	3039992	66463245	1	41123	142068	554118	12643034000	512995	161693	7.082
	2011	250324	3012288	67275390	1	41243	143296	552156	14255958000	510912	161369	7.090
	2012	257144	2985455	68894826	1	41309	141724	545992	15149263000	504683	160563	7.049
	2013	263342	2995711	69706050	1	41933	143006	546425	14619772000	504492	161895	6.978
	2014	272874	2996407	70849049	1	43276	145434	551165	14369843000	507890	165493	6.881
	2015	280922	2978913	66932991	1	43633	146394	554249	15128623000	510616	166515	6.879

(Continued)

Table S1. Continued.

Variable	Year	Sample size	Mean	Standard deviation	Minimum	25%-quantile	Median	75%-quantile	Maximum	Interquartile range	Geometric mean	Geometric stand. dev.
Capital Stock [$\times 10^3$ yen]	1994	854331	95849	3682747	3	3000	10000	15000	1937553152	12000	8131	3.782
	1995	875837	97724	3558007	3	5000	10000	15000	1384222077	10000	9446	3.438
	1996	893955	104166	4196309	1	5000	10000	15000	1937553152	10000	11026	3.029
	1997	914952	108642	4431289	1	6000	10000	15000	1937553152	9000	11174	3.014
	1998	942452	105873	4168308	1	5000	10000	15000	2431711152	10000	11048	3.034
	1999	972305	108356	4547394	1	5000	10000	15000	2642885152	10000	10864	3.057
	2000	993005	118293	5968979	1	5000	10000	15000	2837949000	10000	10813	3.099
	2001	1015856	118754	6096227	1	5000	10000	15000	2987504152	10000	10698	3.129
	2002	1033366	119401	5893300	1	5000	10000	15000	3122575655	10000	10571	3.160
	2003	1042965	114247	4946269	1	5000	10000	15000	3016150666	10000	10480	3.184
	2004	1043259	115878	5009836	1	5000	10000	15000	3016150000	10000	10478	3.215
	2005	1037020	120020	5090766	1	5000	10000	15000	3016150666	10000	10482	3.251
	2006	1033383	119909	4032838	1	5000	10000	15000	1268807877	10000	10452	3.291
	2007	1038135	123609	4104516	1	5000	10000	15000	1268807877	10000	10359	3.349
	2008	1060282	123451	3911395	1	4000	10000	15000	1095543220	11000	10189	3.428
	2009	1096934	125045	8166824	1	3000	10000	15000	7491288000	12000	9792	3.484
	2010	1143646	118700	3972260	1	3000	10000	15000	1118378000	12000	9570	3.632
	2011	1171438	128605	9385173	1	3000	10000	15000	7708783583	12000	9429	3.753
	2012	1187588	130372	9467233	1	3000	10000	14000	7747015342	11000	9249	3.851
	2013	1200282	135279	10301850	1	3000	10000	14000	7800176000	11000	9134	3.928
	2014	1214582	131250	9561387	1	3000	10000	14000	7838998486	11000	9032	4.008
	2015	1222063	131290	9559768	1	3000	10000	14000	7894415052	11000	8949	4.067
